# A guinea fowl genome assembly provides new evidence on evolution following domestication and selection in galliformes

**DOI:** 10.1111/1755-0998.13017

**Published:** 2019-05-05

**Authors:** Alain Vignal, Simon Boitard, Noémie Thébault, Guiguigbaza‐Kossigan Dayo, Valentine Yapi‐Gnaore, Issaka Youssao Abdou Karim, Cécile Berthouly‐Salazar, Nóra Pálinkás‐Bodzsár, Daniel Guémené, Francoise Thibaud‐Nissen, Wesley C. Warren, Michèle Tixier‐Boichard, Xavier Rognon

**Affiliations:** ^1^ GenPhySE INRA, INPT, INP‐ENVT Université de Toulouse Castanet Tolosan France; ^2^ CIRDES Bobo‐Dioulasso Burkina‐Faso; ^3^ Ecole Polytechnique d'Abomey‐Calavi Université d'Abomey‐Calavi Cotonou Bénin; ^4^ UMR DIADE, IRD Montpellier France; ^5^ Université de Montpellier Montpellier France; ^6^ Research Centre for Farm Animal Gene Conservation Gödöllö Hungary; ^7^ Centre INRA Val de Loire, SYSAAF Nouzilly France; ^8^ National Center for Biotechnology Information National Library of Medicine National Institutes of Health Bethesda Maryland; ^9^ McDonnell Genome Institute Washington University School of Medicine St. Louis Missouri; ^10^ Bond Life Sciences Center University of Missouri Columbia Missouri; ^11^ GABI, INRA AgroParisTech Université Paris‐Saclay Jouy‐en‐Josas France

**Keywords:** domestication, genetic selection, genome, helmeted guinea fowl

## Abstract

The helmeted guinea fowl *Numida meleagris* belongs to the order Galliformes. Its natural range includes a large part of sub‐Saharan Africa, from Senegal to Eritrea and from Chad to South Africa. Archaeozoological and artistic evidence suggest domestication of this species may have occurred about 2,000 years BP in Mali and Sudan primarily as a food resource, although villagers also benefit from its capacity to give loud alarm calls in case of danger, of its ability to consume parasites such as ticks and to hunt snakes, thus suggesting its domestication may have resulted from a commensal association process. Today, it is still farmed in Africa, mainly as a traditional village poultry, and is also bred more intensively in other countries, mainly France and Italy. The lack of available molecular genetic markers has limited the genetic studies conducted to date on guinea fowl. We present here a first‐generation whole‐genome sequence draft assembly used as a reference for a study by a Pool‐seq approach of wild and domestic populations from Europe and Africa. We show that the domestic populations share a higher genetic similarity between each other than they do to wild populations living in the same geographical area. Several genomic regions showing selection signatures putatively related to domestication or importation to Europe were detected, containing candidate genes, most notably *EDNRB2*, possibly explaining losses in plumage coloration phenotypes in domesticated populations.

## 1 INTRODUCTION

The helmeted guinea fowl, *Numida meleagris*, belongs to the Galliformes order and the Numididae family. Its natural range includes large parts of sub‐Saharan Africa, from Senegal to Eritrea and from Chad to South Africa, where eight subspecies have been identified (Belshaw, 1985; Crawford, 1990). A ninth subspecies (*N. m. sabyi*), probably extinct today, was present in Morocco (del Hoyo, Elliott, & Sargatal, 1994). Guinea fowl is a sedentary bird, living in flocks (except during the breeding period, where it lives in pairs) mainly in savanna or savanna–bush areas. It is an opportunistic omnivore (Crawford, 1990).

The domestication of this species may have occurred about 2,000 years BP (Larson & Fuller, 2014) in Mali and Sudan where some archaeozoological and artistic data have been found (Serjeantson, 2009), and as the domestic populations are commonly named ‘guinea fowl’, we will use this generic term also to describe the wild species. Guinea fowl was primarily a source of food (Crawford, 1990), but also likely a sentinel for approaching danger (Gifford‐Gonzalez & Hanotte, 2011). This species is also known for its ability to consume parasites such as ticks, and to hunt snakes (Gifford‐Gonzalez & Hanotte, 2011). Interestingly, in the USA an attempt to use guinea fowl to control ticks on cervids (Duffy, Downer, & Brinkley, 1992) was later shown to be ineffective against tick‐borne zoonoses (Ostfeld, Price, Hornbostel, Benjamin, & Keesing, 2006). These uses and the attraction for human settlements (water and food) could be the source of domestication via a process of commensal association (Crawford, 1990; Gifford‐Gonzalez & Hanotte, 2011) as defined by Zeder (2012). Within the village poultry farming conditions, it is a hard‐to‐breed species (Gifford‐Gonzalez & Hanotte, 2011; MacDonald, 1992) with a strong ability to move away and lay far from the farming space. It has a lower productivity than chicken, whose importance has increased rapidly since its introduction in Africa. Despite this, the guinea fowl has been widely dispersed in the Mediterranean world (e.g. Greece, Rome). They practically disappeared from Europe after the fall of the Roman Empire, but returned via Portuguese introduction in the 16th century (Belshaw, 1985; Crawford, 1990) from West Africa.

Today, domestic guinea fowl is still reared in Africa, where it is mainly a village poultry that can constitute a non‐negligible part of the financial and food resources (mainly meat, but also eggs). In these countries, local domestic populations, freely raised around the villages, coexist with the wild populations, providing opportunities for random admixture events. More intensive livestock farming has been developed in some countries since the 1960s, especially with a view towards diversifying meat production. France is the leading producer of guinea fowl, with 75% of European production and 66% of world production in 2010 (Agreste Synthèses – Aviculture, 2011). In 2017, French production was 30,000 tons (Agreste Synthèses – Aviculture, 2018). Selection is essentially performed by two companies based in France and working at the international level, Galor and Hendrix Genetics Turkeys France, subsidiary companies of Groupe Grimaud and Hendrix Genetics, respectively. Until now, a few genetic studies have been carried out to describe genetic diversity in domestic and wild populations using microsatellite (Kayang et al., 2010; Weimann et al., 2016), or mtDNA (Adeola et al., 2015; Walker, Bowie, Ratcliffe, & Crowe, 2004) data. The use of microsatellite markers developed in other Galliforme species such as chicken and quail has very limited value due to sequence amplification problems, and only few specific markers have been developed in guinea fowl (Botchway, Adenyo, Kayang, Hayano, & Inoue‐Murayama, 2013; Kayang et al., 2010).

A whole‐genome sequence assembly of the studied species is now considered as a prerequisite for any large‐scale work involving genomics. Chicken and other major poultry species, such as turkey and the common duck, have benefited from such a resource for several years (Dalloul et al., 2010; Hillier et al., 2004; Huang et al., 2013), and in the case of chicken, several updates of the reference genome have been released, making it one of the best available for vertebrates and a focal point for birds (Warren et al., 2017). Today short‐read sequencing technology (Illumina) allows for the automated production of deep sequence coverage at low cost, and as a result, there has been a rapid increase of the number of bird whole‐genome assemblies available, with at least one representative per bird order. These 48 avian genomes were used for in‐depth analyses of bird evolution (Jarvis et al., 2014; Zhang et al., 2014). To test hypotheses on the origins of avian domestication concerning specifically galloanserae species, we initiated genomics analyses in guinea fowl by producing a first‐generation whole‐genome sequence draft assembly which we then used as a reference for a sequencing study of several populations. In this study, individuals from 12 wild and domestic guinea fowl populations from African and European origins were sequenced as a single DNA pool per population, allowing a description of the population structure and the detection of selective sweeps. We suggest some of these sweeps may result from earlier domestication processes in Africa and others from more recent intense breeding schemes in Europe.

## 2 METHODS

### 2.1 Genome sequencing, contig and scaffold assembly

To minimize genome assembly problems due to polymorphism, candidate individuals were selected after one generation of brother–sister mating within the conservatory g44 inbred domestic line (Galor‐SYSAAF, France). Eleven 21‐day‐old male *N. meleagris* (sample names 19001–19011) were selected, from which blood was sampled and genomic DNA extracted using a high‐salt extraction method (Roussot et al., 2003). In order to use the highest possible DNA quality and quantity from a single sample for the construction of multiple sequencing libraries, individual 19003 was selected, based on its high DNA concentration (1.1 μg/μl) as estimated by a PicoGreen^®^ assay and agarose gel electrophoresis.

Our sequencing plan followed the recommendations provided in the allpaths2 assembler (Maccallum et al., 2009) requiring overlapping paired reads and nonoverlapping mate‐pair reads. All sequences were generated at the McDonnell Genome Institute, Washington University School of Medicine, St. Louis, MO, USA, on the HiSeq2000 Illumina instrument, producing 100 bp reads. Therefore, the overlapping libraries were sized at 180 bp. Two libraries for overlapping paired reads were sequenced in four lanes, five 3 kb mate‐pair libraries in nine lanes and one 8 kb mate‐pair library in a single lane. Details of SRA accessions and quantity of sequence produced for each library and lane are given in Table S1. Low quality and duplicate reads were removed with Picard tools. The combined sequence reads were assembled using allpaths2 software (Maccallum et al., 2009) with default parameters.

### 2.2 Assembling the scaffolds into chromosomes by alignment to the chicken genome

Scaffolds were aligned to the chicken genome (Galgal5) with the lastz software (Schwartz et al., 2003), using the following parameters: (a) step = 30, (b) exact = 40, (c) chain, (d) gapped and (e) format = general:score,name1,start1,end1,length1,name2,start2+,end2+,length,strand for the format of the output file. All other parameters are default. Before assembling the aligned scaffolds into chromosomes, all known interchromosomal rearrangements between chicken and guinea fowl, as documented by Shibusawa et al. (2002) for macrochromosomes, were taken into account. Custom Python and R scripts (Supporting information) were then used in order (a) to sort the Lastz data file in ascending order according to the chromosome coordinates; (b) to create chromosome‐level assemblies joining scaffold sequences in the correct order and orientation, following the sorted Lastz output; and (c) to align the guinea fowl chromosomes thus obtained against the chicken genome for a graphical inspection of the assembly. To remove all contaminating contigs, the genome was screened against the RefSeq chromosomes of nonchordate organisms and contigs with BLAST hits over 98% identity over 100 bases were trimmed or excluded. All contigs <200 bp were removed prior to final assembly submission. To test the quality of the assembly, the aves_odb9 dataset of single copy, orthologous, Avian specific genes from orthodb version 9 (Zdobnov et al., 2017) was selected to check their status (present, duplicated, fragment or missing) with busco version 3.0.2 (Waterhouse et al., 2017) in the Galgal4, Galgal5 and GRCg6a assemblies of the chicken genome and in our NumMel1 guinea fowl assembly. The assembly is publicly available in NCBI Assembly under the name of NumMel1.0 (accession GCA_002078875.2). NumMel1.0 was annotated for gene content using the NCBI Eukaryotic Genome Annotation Pipeline. Same‐species transcripts and proteins available in GenBank, and RNA sequencing (RNA‐Seq) reads available in SRA for six different tissues: uterus (PRJNA383810), pancreas, bursa, bone marrow and hypothalamus (PRJNA168045), spleen, and male and female gonads (PRJNA271731), were aligned to the genome masked for repetitive elements with Windowmasker (Morgulis, Gertz, Schäffer, & Agarwala, 2006), along with the bird, human and *Xenopus* known RefSeq proteins (with the NP_ prefix), the *Gallus gallus* model RefSeq proteins (with the XP_ prefix), and the bird and *Xenopus* GenBank proteins available in the NCBI Entrez Protein database on the day the annotation started (5 June 2017). The gene models’ structures and boundaries were derived from the alignments and complemented with HMM‐based ab initio spans by Gnomons, where the alignments only partially covered open‐reading frames with high enough coding propensity (score of 40). Coding genes were assigned a function based on orthology to human and homology to SwissProt proteins. The final annotation is named *N. meleagris* Annotation Release 100, and its results are summarized in the web report https://www.ncbi.nlm.nih.gov/genome/annotation_euk/Numida_meleagris/100/. A full description of the NCBI gene annotation pipeline was previously published (Pruitt et al., 2014). The genome sequence and the resulting annotation are publicly available for download at NCBI ftp://ftp.ncbi.nlm.nih.gov/genomes/all/GCF/002/078/875/GCF_002078875.1_NumMel1.0/.

### 2.3 Genetic diversity sampling and sequencing

**Figure 1:**
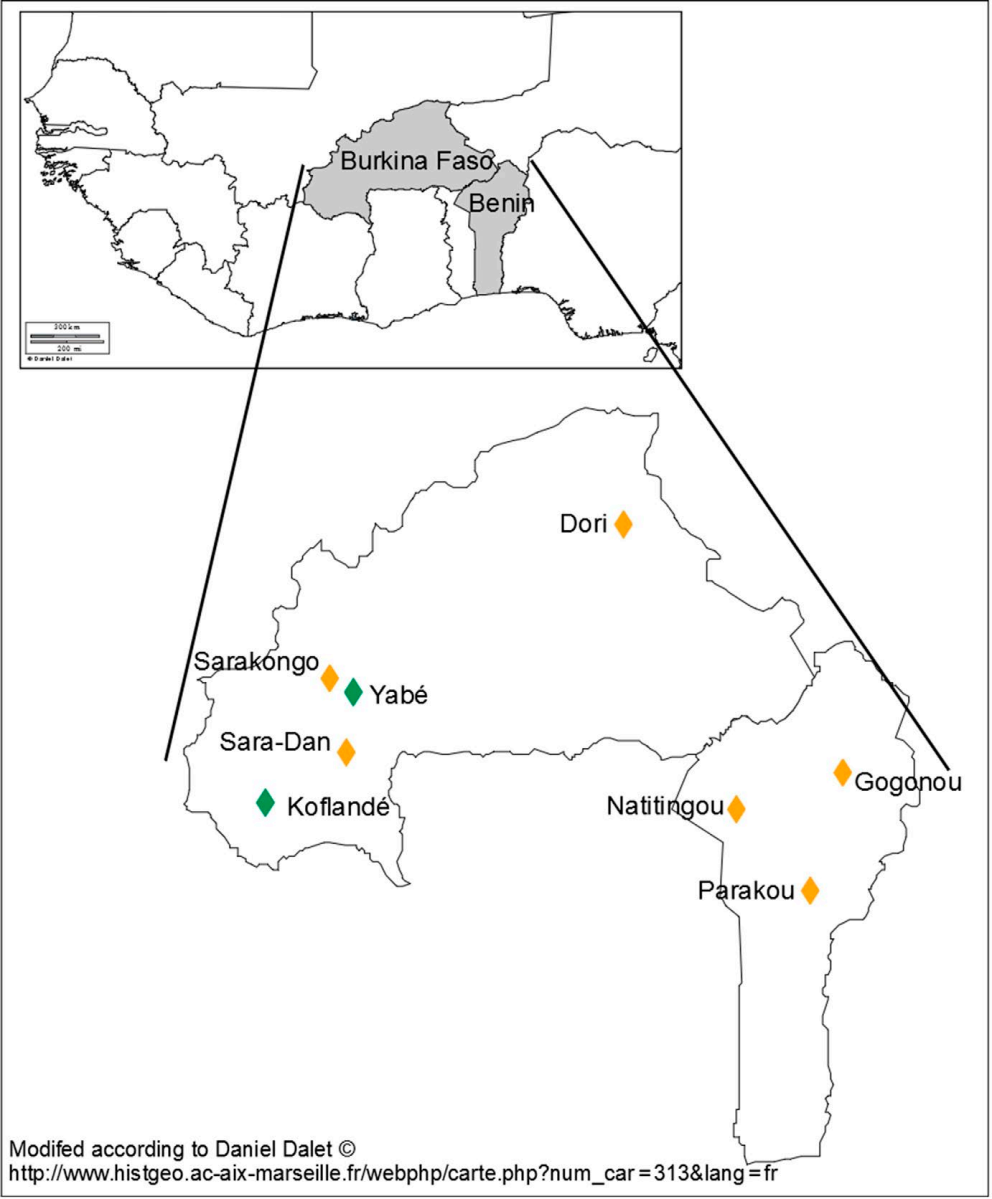
Sampling locations in West Africa. Green: wild populations, orange: African traditional populations [Colour figure can be viewed at wileyonlinelibrary.com]

In order to evaluate genetic diversity in guinea fowl, we sampled individuals from 12 different African and European populations (range 3–30 individuals per population) and sequenced a single DNA pool for each population (Table 1 and Figure 1). Sampled populations included three wild African populations (one from South Africa and two from Burkina Faso), four domestic African populations (three from Burkina Faso and one from Benin) and five domestic European populations (two from Hungary and three from France). Domestic guinea fowl from Benin were sampled in three locations (Figure 1) and pooled for sequencing. Individual pictures were taken for each African domestic individual and for one group of wild guinea fowls from Burkina Faso. Geographical coordinates were registered for each bird together with the name of the village (Table S2). Hungarian samples were traditional guinea fowl populations collected in two conservatories flocks. French samples were collected from two breeding companies. Individuals from the Beghin (BEG‐s) population sample (Grimaud Frères Sélection) were a commercial intercross (between one Beghin line and one Grimaud Frères Sélection line), and those from the two other population samples (GAL‐s and Gri‐s) represented a pool of different selected lines from each company (Galor and Grimaud Frères Sélection).

**Table 1:** Description of the samples used for Pool‐seq analyses and sequencing depth

Population	Type	Nb. individuals	Total reads	Expected depth	Unmapped reads (%)	Duplicate reads (%)	Useable depth
AFS‐w: South Africa	Wild	3	75 444 609	14.51	16.15	2.61	11.57
KOF‐w: Koflandé, Burkina Faso	Wild	8	90 110 060	17.33	10.97	1.58	15.01
YAB‐w: Yabé, Burkina Faso	Wild	8	101 946 710	19.61	9.61	1.22	17.36
SDA‐t: Sara‐Dan, Burkina Faso^a^	Traditional	5	46 946 537	9.03	11.11	1.30	7.85
SKO‐t: Sarakongo, Burkina Faso^a^	Traditional	5	66 238 190	12.74	10.84	1.29	11.11
DOR‐t: Dori, Burkina Faso^a^	Traditional	5	39 031 810	7.51	12.58	1.81	6.36
BEN‐t: Benin	Traditional	15	79 256 741	15.24	8.96	0.91	13.67
GOD‐t: Godollo, Hungary	Traditional	30	85 677 433	16.48	11.19	1.60	14.23
HAR‐t: Hortobagy, Hungary	Traditional	30	92 634 659	17.81	12.45	1.84	15.08
BEG‐s: Beghin, France	Selected	12	77 041 946	14.82	12.02	1.66	12.64
GAL‐s: Galor, France	Selected	29	192 437 214	37.01	13.35	2.79	30.44
GRI‐s: Grimaud, France	Selected	20	201 393 063	38.73	13.10	2.82	31.92

a Due to low sequencing depth, these three populations were merged into one Burkina Faso population, named BUR‐t, in genomic scans for selection analyses. For wild and traditional samples, the sampling location is indicated. The breeding company is given for selected populations.

Paired‐end sequencing with 100 bp reads was performed at the Genotoul Get‐platform in Toulouse, France, on Illumina HiSeq 2000 and 2500 instruments, following the manufacturer's protocols for library preparations. Each library from one DNA pool was sequenced in three different runs to eliminate possible run effects. Sequences for this project have been deposited in the Sequence Read Archive (SRA) at www.ncbi.nlm.nih.gov/sra under the project accession PRJNA496587.

Sequence reads were aligned to the genome reference with BWA mem version 0.7.12 (Li, 2013), using options –M –R; the resulting SAM file was then sorted and duplicate reads marked with picardtools version 1.88 SortSam.jar and MarkDuplicated.jar (http://broadinstitute.github.io/picard/); BAM files corresponding to the same population were merged with samtools version 1.3 merge (Li et al., 2009), and sequences were realigned around indels using gatk version 3.3.0 GenomeAnalysisTK.jar −T IndelRealigner (McKenna et al., 2010). A pileup file was created for each population with Samtools mpileup (Li et al., 2009).

### 2.4 Variant calling and allele frequency estimation

Within each pool, the frequency of the minor allele was estimated for all genomic positions that were covered by at least five reads, using pool‐hmm (Boitard et al., 2013), https://forge-dga.jouy.inra.fr/projects/pool-hmm. This software is dedicated to the analysis of Pool‐seq data. For each genomic position, it computes the likelihood of all possible allele frequencies, accounting for two important features of Pool‐seq experiments: (a) the variance of sequencing depth and of individual contributions to the pool along the genome, and (ii) the sequencing error probabilities. Based on these likelihoods, the genome‐wide allele frequency spectrum is computed using a random sample of genomic positions (option only‐spectrum), and the software returns for each genomic position the allele frequency with the highest posterior probability (option estim). In our analysis, the proportion of genomic positions used to compute the allele frequency spectrum was set to 0.001 (option −R) according to the author's recommendations, and the starting value for the population mutation rate (option −t) was the default value 0.005.

Allele frequency files obtained for each pool were merged using the python script estim2freq.py, available from the pool‐hmm webpage. This provided 80,779,963 chromosomal variants with at most two alleles (when 3 or more alleles were observed at a given position, only the two most frequent were kept). Monomorphic variants and variants with missing data in at least one of the pools were then removed using r (Supporting information), leading to a final set of 10,205,115 filtered variants. Variants located on chromosome Z, contig LGE64 or the mitochondrial DNA were also obtained, but were not included in following analyses.

In genomic scans for selection (see below), the three domestic populations from Burkina Faso were considered jointly, providing a population named BUR‐t. For this purpose, we applied the procedure described above after having merged the three pileup files corresponding to these pools.

### 2.5 Diversity measures

Expected heterozygosity in a given population was computed as the average of 2*p*(1−*p*) over the 10,205,115 filtered variants, where *p* is the minor allele frequency. A correction factor (1−1/(2*n*)) was applied in order to account for the number of individuals n within each pool. For each population, the Watterson *θ* was computed directly from the pileup file using popoolation (Kofler, Pandey, & Schlötterer, 2011), https://sourceforge.net/p/popoolation/wiki/Main/. This software provided an estimation for nonoverlapping 100 kb windows all along the genome. We averaged these values, accounting for the number of genomic positions effectively used within each window (positions covered by less than five, or more than 100, reads with sufficient quality were not considered by popoolation). In order to evaluate the influence of unequal genome coverage between pools, we compared the estimates obtained from raw data with those obtained after subsampling all populations and positions at a uniform 10 × coverage, focusing on a small part of the genome (Chromosome 8). Principal component analysis was performed with the *pca* function of the r *mixOmics* library (Lê Cao, González, & Déjean, 2009), http://mixomics.org/. This function was applied to the allele frequency file described above.

### 2.6 Selection signatures, within‐population approach

Selective sweeps within each population were detected using the *pred* option of pool‐hmm. This command implements the Hidden Markov Model (HMM) approach originally proposed by Boitard, Schlotterer, and Futschik (2009) and adapted to Pool‐seq data by Boitard, Schlotterer, Nolte, Pandey, and Futschik (2012). The objective of this approach is to identify genomic regions showing an excess of rare alleles, compared to what is expected from the genome‐wide allele frequency spectrum. The model includes three possible hidden states, ‘Neutral’, ‘Intermediate’ and ‘Selection’, which are associated with different allele frequency spectra, and the objective is to predict which genomic regions are in the state ‘Selection’, based on observed allele frequencies. In order to account for the high uncertainty associated with allele frequency estimations obtained from Pool‐seq data, the extension of Boitard et al. (2012) includes an integration over all possible allele frequencies at each genomic position (as already mentioned above, the first step is to compute the likelihood of these frequencies). This allows exploiting information from all genomic positions, while putting more weight on those with higher coverage or higher read qualities. In our analyses, the transition probability to the hidden state ‘Selection’ of the HMM (parameter *k*) was set to 0.000001.

After running pool‐hmm within each pool, we focused on three specific types of sweep regions: (a) potential domestication sweeps, which were detected in at least six (out of seven) domestic populations while showing no significant signal in any of the three wild populations; (b) potential European sweeps, which were detected in at least four (out of five) European populations while showing no significant signal in any of the five wild and domesticated African populations; and (c) potential ‘production’ sweeps, which were detected in the three populations managed with strong artificial selection criteria, while showing no significant signal in any of the seven other populations.

### 2.7 Selection signatures, multipopulation approach

Genomic regions showing an excess of genetic differentiation between populations, compared to what is expected under neutral evolution, were detected using the local score approach of Fariello et al. (2017). This approach proceeds in two steps. First, a *p*‐value measuring the evidence for selection is computed independently for all observed variants. Second, regions with an excess of low *p*‐values are detected using the statistical local score theory. More precisely, (a) each *p*‐value is converted into the score −log_10_(*p*)−*ε*, where *ε* is a threshold to be set by the user, (b) a cumulated score called the Lindley process is computed for each genomic position, and (c) local maxima of this cumulated process are considered as candidate regions, because they correspond to regions with an excess of low *p*‐values. Compared to sliding window approaches, the local score approach avoids defining arbitrary sliding windows and allows quantifying the statistical evidence of detected regions, that is the significance level of local maxima of the Lindley process.

Following Fariello et al. (2017), we computed single marker *p*‐values using the FLK test (Bonhomme et al., 2010), implemented in the hapflk software, https://forge-dga.jouy.inra.fr/projects/hapflk. The FLK statistic is an extension of the classical FST statistic that accounts for differences of effective population size among populations, and for the hierarchical structure of populations. In this approach, the neutral evolution of allele frequencies is modelled by a population tree, whose branch lengths correspond to drift units (the length of a branch is the probability that two alleles sampled at the bottom of this branch descend from the same allele at the top of this branch). This neutral tree is first estimated using genome‐wide data, and the deviation from this tree is then tested for each SNP by the FLK statistic. If migration between populations and genetic drift is not too high, the expected distribution of FLK under neutral evolution is a chi‐square with *n* − 1 degrees of freedom, where n is the number of observed populations. Thus, computing this statistic and the associated *p*‐values for all variants in a sequence is straightforward. We computed FLK for a set of nine populations: because of small sample sizes, the three domestic populations from Burkina Faso were merged into a single one named BUR‐t, and AFS‐w was removed, although it was still used to root the population tree. For this analysis, we focused on SNPs with a minor allele frequency >10% in at least one the nine analysed populations.

**Figure 2:**
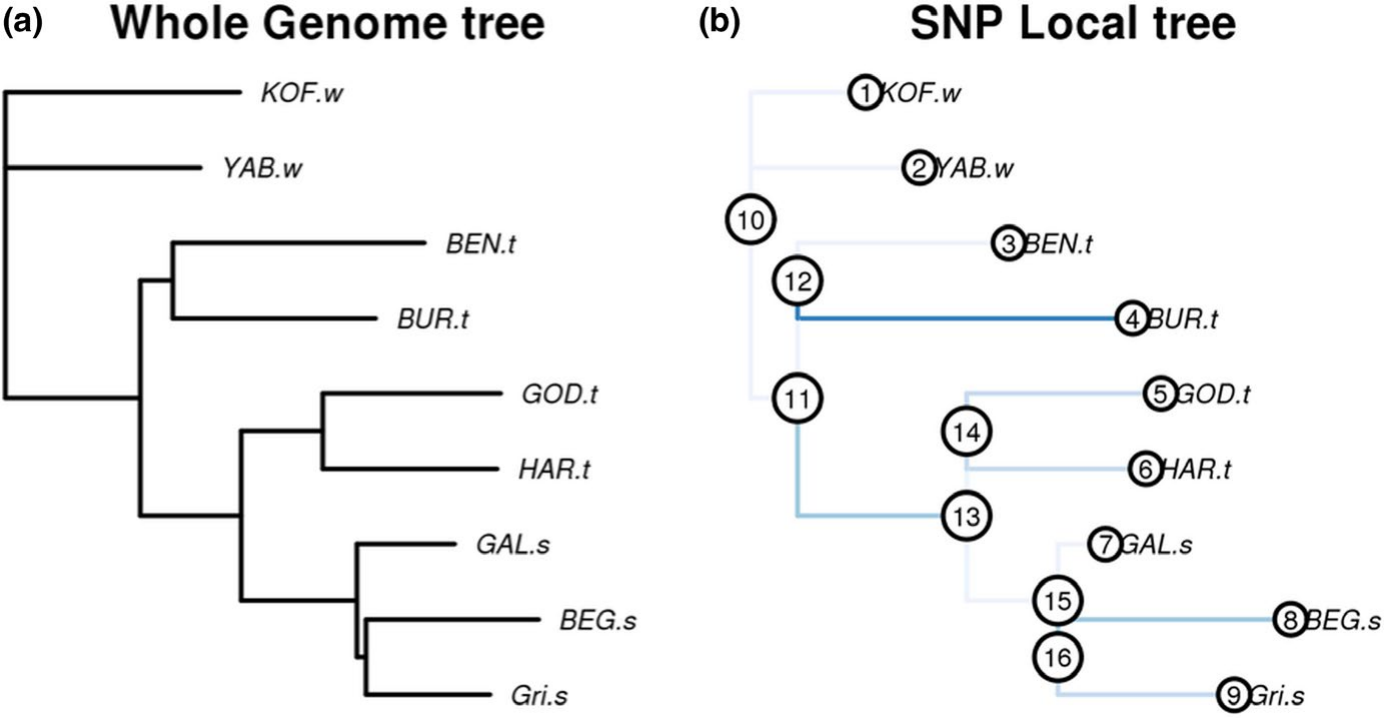
Population tree of wild, traditional and selected populations of guinea fowl. (a) Whole‐genome population tree estimated by the FLK approach (Bonhomme et al., 2010) from genome‐wide allele frequencies in 10 populations (the three domestic populations from Burkina Faso were merged). The length of each branch corresponds to the amount of drift accumulated on this branch, which is roughly equal to *t*/*N*, where *t* is the evolution time (in generations) and *N* the effective population size. The wild population from South Africa was used as outgroup to root the tree. (b) Local population tree corresponding to one of the eight regions detected under selection by the local score approach of Fariello et al. (2017). This region is located on chromosome 1, from 162,467,697 to 163,260,131 bp. Branches with blue colour indicate differing length when compared to the whole‐genome tree, with higher intensities corresponding to most significant differences. The branch between nodes 11 and 13, which leads to all European populations, is significantly longer than in the genome‐wide tree, suggesting a selection event related to importation into Europe. The branch leading to BUR‐t is also significantly longer, suggesting that the region may also be related to domestication. However, the topology of the tree indicates that the alleles selected in BUR‐t differ from those selected in Europe [Colour figure can be viewed at wileyonlinelibrary.com]

We applied the local score to FLK *p*‐values using a score function with *ε* = 2, which means that we cumulated *p*‐values below 0.01. Fariello et al. (2017) indicated that this choice was in principle preferable to *ε* = 1 for the detection of old selection events, as this gives more weight to short segments with high *p*‐values, compared to long segments with moderate *p*‐values. In their simulations, selection started 20 or 40 generations ago, which is younger than what we targeted here when studying the effects of domestication or importation to Europe. Detection thresholds for each chromosome were computed for a type I error rate of 5%, using the Gumbel assumption and the re‐sampling procedure proposed by the authors in the case where the distribution of *p*‐values under the null hypothesis (neutral evolution) is nonuniform. Indeed, this was the case in our analysis, because the assumption of a chi‐square distribution for FLK did not exactly hold, due to the high genetic drift observed between populations (Figure 2a).

For all detected regions, we built a local population tree and identified the branches whose length was significantly longer than in the genome‐wide tree, using python and R scripts provided on the hapflk webpage. As indicated by Fariello, Boitard, Naya, SanCristobal, and Servin (2013), this procedure allows identifying the population(s) under positive selection in the region.

### 2.8 Functional analysis of candidate regions

For each detected region under selection, the genes and the potential causal variants included in the region were listed based on the GFF annotation file available on the NCBI website: ftp://ftp.ncbi.nlm.nih.gov/genomes/all/GCF/002/078/875/GCF_002078875.1_NumMel1.0/. For a given category of selection event (domestication, importation to Europe or intensive production), we only considered variants having one of the two alleles at high frequency (0.75 or more) in all selected populations and at low frequency in all nonselected populations (0.25 or less). AFS‐w was not considered in this analysis, because allele frequencies in this population could not be estimated with sufficient precision, due to the very small sample size. The functional analysis of candidate variants was performed using snpeff, version 4.2 (http://snpeff.sourceforge.net/index.html). Enrichment in specific Gene Ontology categories was tested using g:profiler (Reimand, Kull, Peterson, Hansen, & Vilo, 2007).

## 3 RESULTS

### 3.1 Genome assembly

Total sequence genome input coverage on the Illumina HiSeq 2500 instrument was ~160 × (62 × overlapping fragments, 92 × of 3 kb and 6 × of 8 kb paired‐end reads), estimated using a genome size of 1.04 Gb (Table S1). The assembled male genome NumMel1.0 is made up of a total of 2,739 scaffolds (including single contig scaffolds) with a scaffold N50 length of 7.8 Mb (contig N50 length is 234 kb). The assembly sequence size is 1.04 Gb with only 3.8% (38 Mb) not assigned to chromosomes, and the NumMel1.0 assembly metrics were comparable to previous assemblies of Galliformes (Table S3). The quality of the assembly was also estimated by testing the presence of the 4,915 single copy orthologous Avian specific genes from orthodb version 9 with the busco version 3.0.2 pipeline (Waterhouse et al., 2017). When compared with the three last chicken assemblies, the percentage of missing, fragmented or duplicated genes is similar (Table S4). Finally, our estimate of total interspersed repetitive elements based on masking with Windowmasker (Morgulis et al., 2006) was 19.5% genome‐wide.

**Figure 3:**
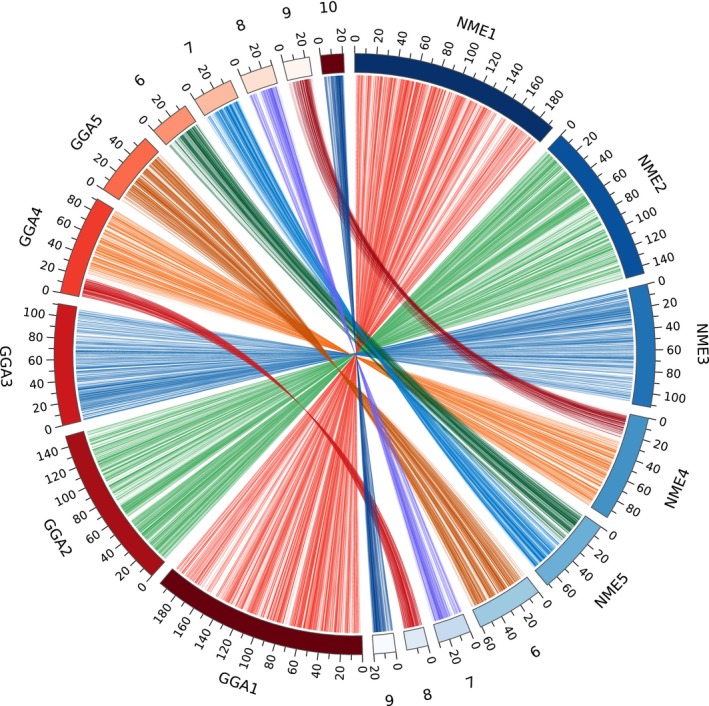
Circos plot comparing the genome alignments of guinea fowl chromosomes 1–9 to chicken chromosomes 1–10. Left (red): chicken chromosomes from the GRCg6a assembly; right (blue): guinea fowl chromosomes from the NumMel1.0 assembly. Alignment was done with the last software as described in Frith and Kawaguchi (2015) [Colour figure can be viewed at wileyonlinelibrary.com]

Guinea fowl scaffolds were aligned to the chicken Galgal5 genome assembly for obtaining a chromosome‐scale scaffold assembly. Chicken and guinea fowl karyotypes are typical of avian genomes, with a few large chromosomes (macrochromosomes) and a much larger set of smaller chromosomes (microchromosomes). The chicken karyotype is composed of 38 pairs of autosomes plus the Z and W gonosomes, and only the ten largest autosomes can be identified by classical cytogenetics methods and are usually referred to as the macrochromosomes (Ladjali, Tixier‐Boichard, & Cribiu, 1995). Attribution of scaffolds to *N. meleagris* chromosomes was done by taking into account the cytogenetic rearrangements described by Shibusawa et al. (2002), and as a result, guinea fowl chromosome NME4 corresponds to chicken chromosome GGA9 and GGA4q; NME5 to GGA6 and GGA7; NME6 to GGA5 and NME7 to GGA8 (Shibusawa et al., 2002). Alignments of chicken and guinea fowl macrochromosomes are presented Figure 3. The remaining guinea fowl microchromosomes are very small, and until now, the status of their nomenclature in comparison with chicken had not yet been considered. Therefore, we decided to align NME8 with GGA4p, and for the remaining microchromosomes, NMEn corresponds to GGAn+1 (Figure 3 and Figure S1). The exact number of microchromosomes in guinea fowl has not been determined to date (Shibusawa et al., 2002), but all the chicken chromosomes having assigned sequence in Galgal5 (six chicken microchromosomes have no sequence) have some sequence similarity to guinea fowl sequence, including the small chicken linkage groups LGE64 (Figure S1). Thirty‐eight Mb of assembly scaffolds could not be attributed to chromosomes, in part due to the missing microchromosomes in Galgal5. In total, the NCBI Eukaryotic Genome Annotation Pipeline identified and annotated 16,101 protein‐coding genes and 43,227 protein models in the *N. meleagris* genome (Table S5), which is in line with other assembled and annotated Galliformes, and suggests the gene representation is sufficient for all analyses described herein.

### 3.2 Genetic diversity

**Figure 4:**
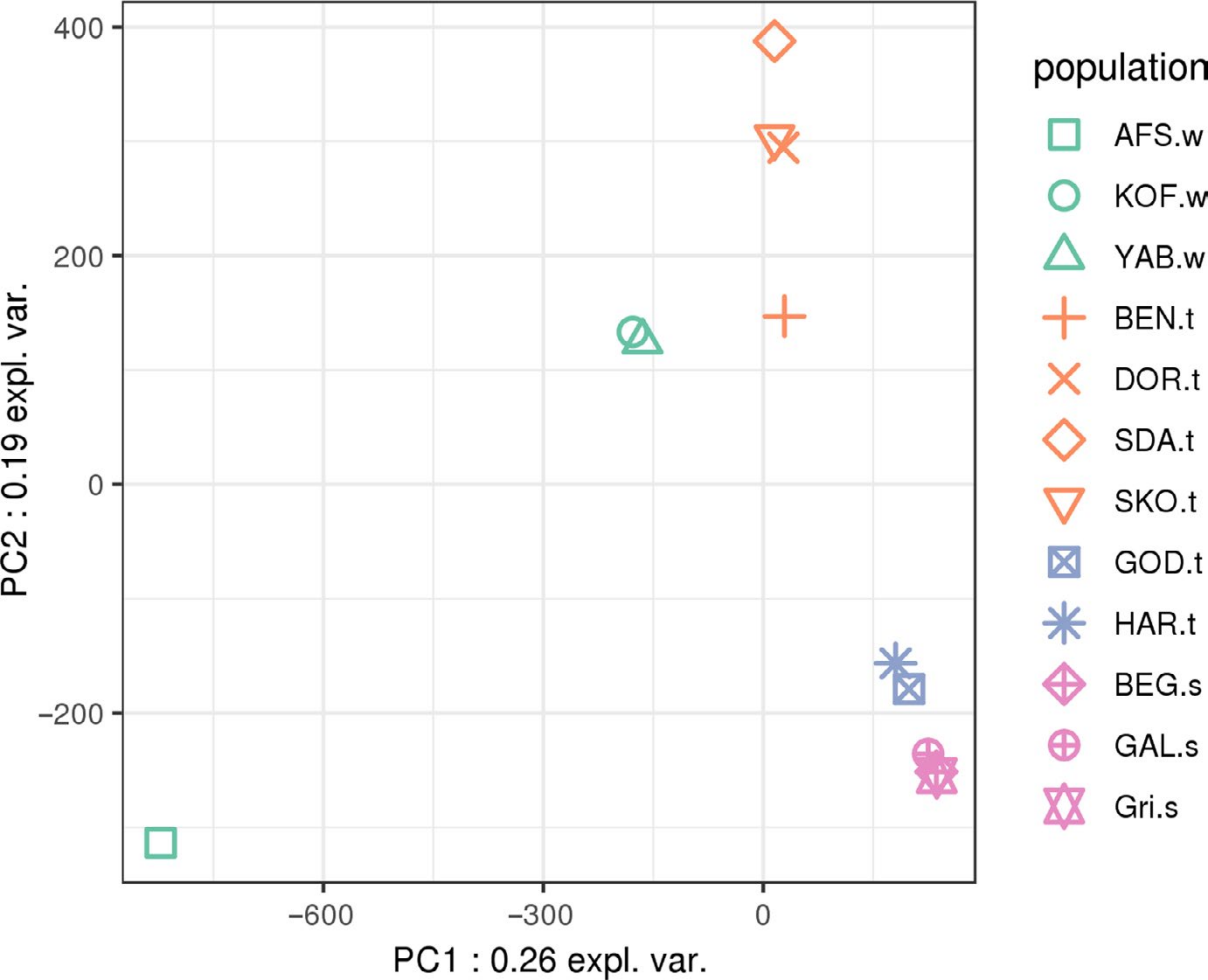
Principal component analysis on allele frequencies. For the complete population names, see Table 1 and Figure 1 for populations sampled in West Africa. Green: wild populations; orange: African traditional populations; blue: European traditional populations; pink: European selected populations from breeders [Colour figure can be viewed at wileyonlinelibrary.com]

To capture the overall structure of this genetic diversity, we first performed a principal component analysis (PCA) of population allele frequencies (Figure 4). The first axis of this analysis, which explained 26% of the variance of our data set, opposed the wild population from South Africa to all other populations, including both wild populations from West Africa and the domestic ones. Interestingly, among West African populations, the distinction between wild and domestic populations was more determinant than geographic effects: the KOF‐w and YAB‐w wild populations were almost overlapping, although the sampling location of YAB‐w is closer to that of domestic populations (SDA‐t and SKO‐t). The second axis, which explained 19% of the variance, opposed European populations to West African populations (either wild or domestic). Focusing on European populations, PC2 also showed the difference between traditional populations from Hungary and more selected populations from France.

**Figure 5:**
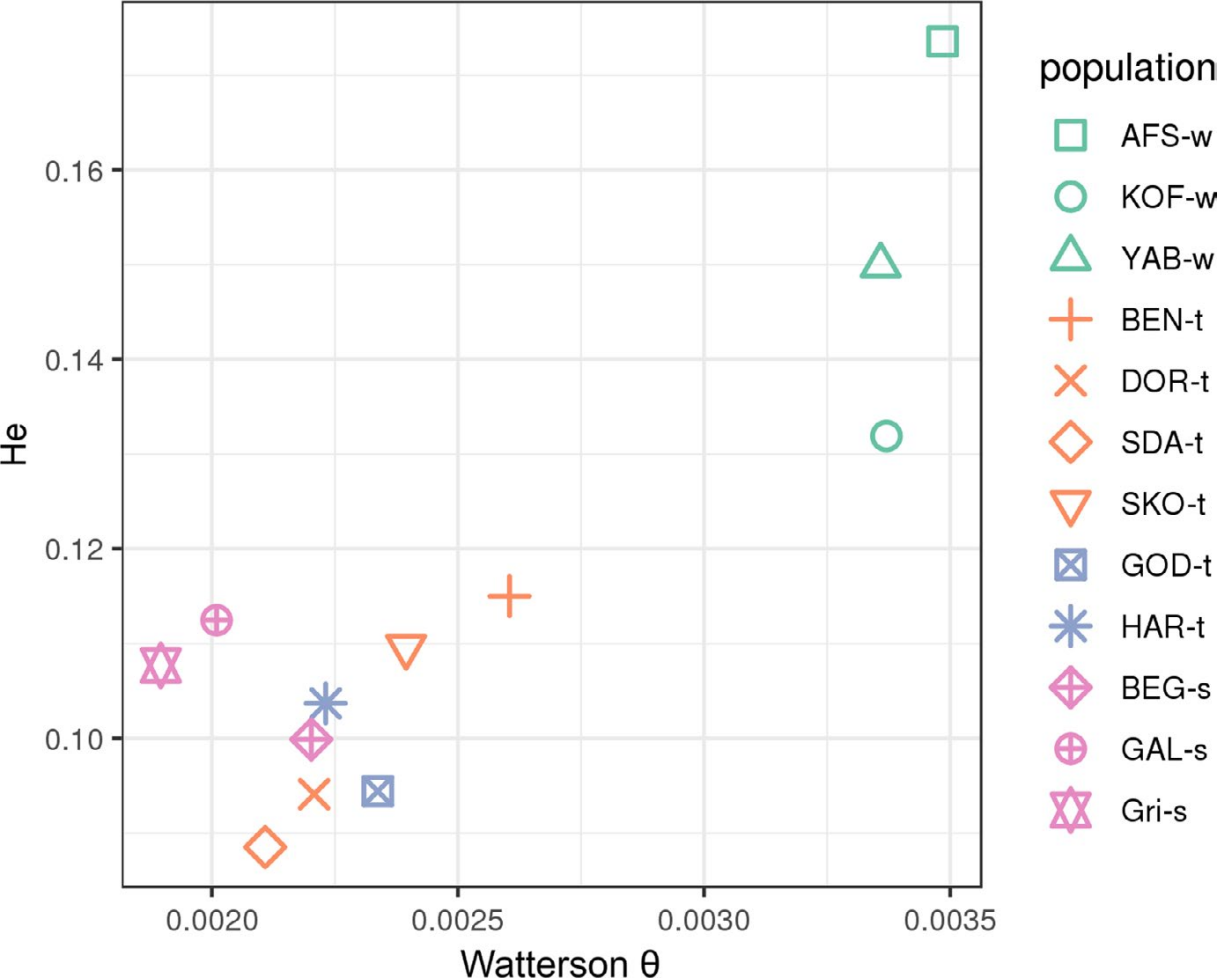
Genetic diversity estimated by Watterson *θ* (*x*‐axis) or average heterozygosity among bi‐allelic variants (*y*‐axis) for each population. Population names and colours as in Figure 3 [Colour figure can be viewed at wileyonlinelibrary.com]

We next measured genetic diversity in each population, using two different estimators: one based on the number of observed variants (Watterson *θ*) and the other on expected heterozygosity (Figure 5). The two measures lead to a slightly different ranking of the breeds, but both confirmed the significantly larger diversity of wild populations, consistent with the idea that domestication has led to the reduction of effective population size. We also checked that this larger diversity of wild populations was not an artefact due to unequal coverage between breeds, see the discussion for more details.

The conclusions above concerning genetic structure and diversity were confirmed when fitting a population tree model to the observed data (Figure 2a). Note that, when building the population tree of Figure 2a, the three populations from Burkina Faso were merged into a single group. Indeed, allele frequencies in these populations cannot be estimated accurately, due to the small sample sizes (five individuals per pool). However, Figures 4 and 5 indicate that these three populations are relatively homogeneous, so we considered them as a single group for this analysis, as well as the selection scans described below. First, this tree showed several hierarchical levels that were consistent with the PCA. At the first level, one subtree including all domestic populations diverged from KOF‐w, YAB‐w and AFS‐w (the latter being the root of the tree). At the second level, the domestic subtree was divided into one African and one European subtree. Finally, at the third level, the European subtree was divided into a French subtree and a Hungarian subtree. Secondly, important differences of branch length were observed between populations. In a pure drift model (i.e. assuming that all genetic variants were already present at the root of the tree), these branch lengths are, approximately, inversely proportional to effective population sizes. Thus, we can conclude from Figure 2a that effective population size is larger in wild populations than in domestic populations, consistent with the results of Figure 5. To a lower extent, effective population size is also lower in European populations than in African populations.

### 3.3 Selection signatures

We next investigated if the serial founder events associated with domestication, importation to Europe and selective breeding, as outlined above, were associated with adaptation at some specific loci. We looked for such loci using two different approaches.

First, we detected selective sweep signatures within each population following the approach of Boitard et al. (2012). Thousands of candidate genomic regions showing reduced genetic diversity (i.e. under selection) were detected across all ten analysed populations. We then considered three categories of populations (wild, traditional and selected) and merged the regions detected across populations of the same category. As a result, five were potentially related to domestication, as they were detected in at least six (out of seven) domestic populations, while showing no significant signal in any of the three wild populations (Table S6). Similarly, 31 regions were potentially related to the importation into Europe (detected in at least four out of five populations) (Table S7), and 64 were potentially related to recent selection for production traits (detected in all three breeder's populations) (Table S8). Allele frequency patterns in two detected regions, one related to domestication and the other to importation into Europe, are illustrated in Figure S2. The total genome coverage of the regions detected is 2.42, 5.89 and 8.59 Mb, respectively, for domestication, importation to Europe and commercial selection. This represents a strong enrichment compared to the expected genome coverage in each category (6.47, 817.58 and 742.36 kb, respectively), considering the proportion of the genome covered by sweeps for each population.

Secondly, we detected genomic regions with outlier genetic differentiation among populations, following the approach of Fariello et al. (2017). This approach computes a *p*‐value measuring the evidence for selection for all observed variants and looks for genomic regions with an excess of low *p*‐values, based on the statistical local score theory. In this approach, we account for linkage disequilibrium (LD) between markers when detecting selection, despite the fact that individual genotypes cannot be observed from Pool‐seq experiments. Eight regions were detected (Table S9 and Figures S3–S5), and two of these regions overlap with domestication signatures detected by the within‐population approach, and one overlaps with a European signature. Local population trees in the five other regions suggest that three of them are also related to domestication or importation into Europe (Figure 2b). Altogether, these three regions cover 1.14 Mb.

**Figure 6:**
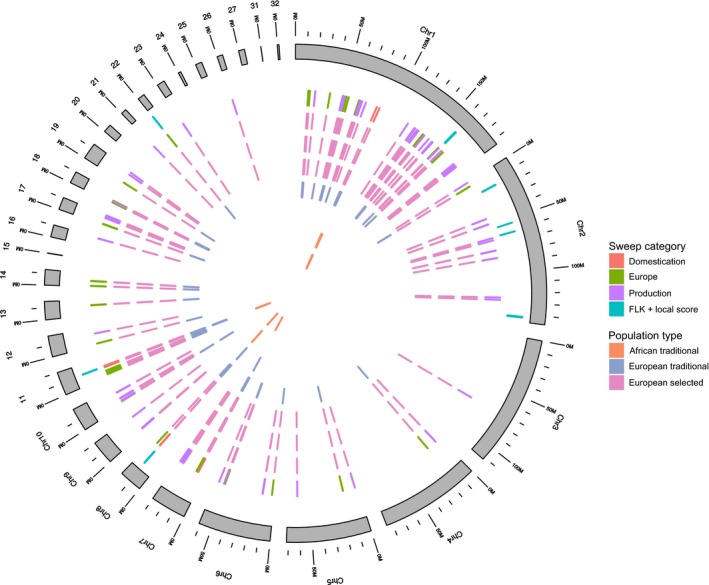
Overview of selection signatures in the genome. Outer grey circle: guinea fowl chromosomes; then going inwards: multipopulation approach selection signatures; within‐population selection signatures related to domestication, importation to Europe and to selection for production traits; exact populations where a selective sweep is detected for each of these regions. One circle relates to a different population, in the following order : the three European selected populations (in pink), the two European traditional populations (in blue) and the two African traditional population from Benin and Burkina Faso (in orange) [Colour figure can be viewed at wileyonlinelibrary.com]

By combining the above within and between population analyses, we obtained 103 regions potentially related to domestication, importation into Europe or commercial selection, covering a total of 1.72% (18 Mb) of the genome (Figure 6). To refine our selection signal, we only considered the intersection of the regions detected for each population within each of the three categories: domestication, import in Europe and intense selection. In the process, some regions previously detected were eliminated and some others were split into several subregions. A total of 114 regions remained (Table S10), covering altogether 0.59% (6.22 Mb) of the genome in which, if distributed randomly, we can expect to find 130 coding or noncoding genes. A total of 223 genes were detected altogether, which is almost twice the expected, suggesting an enrichment towards gene‐rich regions in our results. Among these genes, 122 had gene ontology information that was used to investigate whether enrichment for specific Gene Ontology (GO) terms occurred. No significant results were found. We also investigated potential enrichment for GO terms within each of our three categories of selection signatures: domestication, importation in Europe and commercial selection independently, but again found no significant enrichment for GO terms.

### 3.4 Strong candidate regions and genes

Among the 223 genes found in the regions detected, we narrowed our choice to those possibly having undergone genetic selection by investigating the presence of SNPs with extreme allele frequencies. To this end, only genes having at least one marker for which one of the two alleles was at high frequency (0.75 or more) in all selected populations, while having a low frequency (0.25 or less) in all nonselected populations, were finally kept. All SNPs discussed in the text further down refer to these specific ones. A final list of such 58 genes could be defined, 25 of which have a gene name in the genome annotation (Table 2 for the 25 genes with annotation and Table S11 for the complete list of 58 genes). The number of SNPs detected in genes ranges from one for 14 of the genes, to 88 in *MYO3A*. The 58 genes were grouped together according to their location on the genome, based on the first 103 regions detected, giving a total of 22 candidate regions. A few of these clearly stand out, as they present a high density of SNPs in several genes, such as the region around 15.5–15.7 Mb on chromosome 2 containing six genes including the annotated genes *MYO3A*,* GAD2* and *APBB1IP*; around 11.1–11.5 Mb on chromosome 8 with 15 genes including *TMLHE*,* SPRY3* and *VAMP7* and around 10.6–11.1 Mb on chromosome 11 with seven genes including *UROC1*,* CHCHD4*,* SLC6A6* and *GRIP2* (Table S11). Interestingly, the two regions on chromosomes 8 and 11 were detected by both the within and between population approaches. *PAPPA2* is the only known gene outside these three regions having a high number of SNPs with 27 found and is the only gene detected in a small region around 7.3 Mb on chromosome 7. *SLC41A2* on chromosome 1 around 55.0 Mb contains eight SNPs. All other annotated genes detected contain a lower number of SNPs (Table 2).

**Table 2:** Genes in selected regions with SNPs having extreme frequencies

Gene	NbSNPs	Chrom	GeneStart	GeneEnd	Category
*LHFPL3*	3	Chr1	13 248 980	13 487 001	Europe
*MGAT4A*	1	Chr1	130 175 196	130 249 278	Europe_selected
*TM9SF2*	5	Chr1	142 182 965	142 210 445	Europe
*DLG2*	1	Chr1	187 210 034	188 229 567	Europe_selected
*HEBP1*	1	Chr1	48 924 089	48 930 477	Europe_selected
*ALDH1L2*	2	Chr1	55 024 345	55 053 990	Europe
*C1H12orf45*	2	Chr1	55 054 706	55 059 339	Europe
*SLC41A2*	8	Chr1	55 059 507	55 110 715	Europe
*SH2D1B*	7	Chr1	85 478 336	85 527 878	Domestication
*APBB1IP*	52	Chr2	15 600 834	15 662 526	SL/Europe
*GAD2*	46	Chr2	15 731 592	15 766 219	SL/Europe
*MYO3A* a	88	Chr2	15 766 389	15 876 773	SL/Europe
*DSP*	1	Chr2	62 387 877	62 425 445	SL/Europe
*DOCK10*	1	Chr4	15 717 965	15 934 446	Europe
*KALRN*	1	Chr5	61 774 635	62 233 944	Europe_selected
*PAPPA2* a	27	Chr7	7 327 041	7 402 471	Domestication
*TMLHE*	28	Chr8	11 124 661	11 141 814	Domestication
*SPRY3*	15	Chr8	11 148 808	11 159 454	Domestication
*VAMP7*	13	Chr8	11 300 284	11 318 354	Domestication
*LOC110403465* a (*EDNRB‐like*)	11	Chr8	11 281 712	11 293 849	Domestication
*UROC1*	1	Chr11	10 607 292	10 682 957	Domestication
*CHCHD4*	2	Chr11	10 697 355	10 706 112	Domestication
*SLC6A6*	37	Chr11	10 833 746	10 946 137	Domestication
*GRIP2*	12	Chr11	10 948 291	11 169 922	Domestication
*MATR3*	1	Chr12	1 741 296	1 767 449	Europe
*TYW1*	3	Chr18	825 186	903 045	Europe

a *MYO3A, PAPPA2* and *LOC110403465 (EDNRB‐like)* have one missense polymorphism. *LOC110403465* has no direct annotation, but was included here as potentially interesting due to its missense polymorphism. NbSNPs: SNPs within the gene annotation boundaries having extreme allele frequencies differences between the wild and domestic populations, Chrom: chromosome, GeneStart: position of the beginning of the gene in the assembly, GeneEnd: position of the end of the gene in the assembly, Category: category of the selection signature, related to domestication, importation in Europe or intensive selection. SL/Europe: detected only by the multipopulation approach.

Out of the 22 regions discussed above, three harbour a gene (*MYO3A*,* PAPPA2* and *LOC110403465*) with a polymorphism referred to as having a moderate effect (missense or splice variant) by SNPeff. The first missense polymorphism at position chr7:7,353,449 bp in *PAPPA2* is Met1350Thr and the allele frequencies are as follows: KOF‐w = 0.0; YAB‐w = 0.0 and AFS‐w = 0.17 for the three wild populations and 1.0 for all other populations, except BEN‐t, for which the data are missing. The second missense polymorphism at position chr2:15782251 bp in *MYO3A* is Ser1264Ala, and the allele frequencies are 0.0 in all three wild populations, 1.0 in all five European populations, 0.67 in BEN‐t and 0.87 in BUR‐t. Eight polymorphisms having low effect according to SNPeff (mostly synonymous variants) were also detected in this candidate region on chromosome 2, including three in *MYO3A* (Table S12). The third missense polymorphism at position chr8:11284280 in *LOC110403465* is Thr32Ser, and the allele frequencies are 1.0 in all domesticated populations, and 0.0 in the KOF‐w and YAB‐w populations. Six polymorphisms having low effect were also detected in the region on chromosome 8 (Table S12). Although no specific gene name is attached to *LOC110403465* for guinea fowl in the NCBI Genome Data Viewer, the description in the full report mentions ‘endothelin B receptor‐like’. This was confirmed by a protein BLAST search against the GenBank nonredundant protein database, showing 70% identity over 75% of the guinea fowl sequence with the human endothelin receptor type B isoform protein, coded by the *EDNRB* gene.

## 4 DISCUSSION

In terms of scaffold and contig sizes, our guinea fowl genome assembly shows quality metrics that are quite comparable to those of other Galliformes available to date, such as quail and turkey. Moreover, the assembly continuity, that is ungapped sequence, obtained is superior to most other sequenced and assembled avian genomes using short‐read input (Zhang et al., 2014). Also, the number of genes detected when compared to previously annotated bird species is very similar. To assemble the scaffolds at the chromosome level, we took advantage of the high degree of karyotype conservation observed between gallo‐anseriformes including microchromosomes (Fillon et al., 2007) and used prior knowledge on the major rearrangements observed between chicken and guinea fowl macrochromosomes (Shibusawa et al., 2002). Once the guinea fowl scaffolds were aligned to the chicken genome, we built reliable chromosomal assignments, although we were limited in the number of intrachromosome rearrangements observed, as only those happening within scaffolds can be detected. Only 13 intrascaffold rearrangements were found in our analysis, but certainly an improvement of sequence continuity using long‐read sequencing technology and optical mapping should allow for the detection of more rearrangements in the future.

The natural range of guinea fowls includes large parts of sub‐Saharan Africa, with eight subspecies. Wild samples in our study involve two of them, *N. m. galeata* (Burkina Faso) and *N. m. coronata* (South Africa), which appear clearly differentiated (Figure 4). Domestic (West African and European) populations seem to be more related to the West African wild than to the South African ones. This agrees with previous data (Larson & Fuller, 2014), although very poor archaeozoological data have been found to strictly prove this origin of domestication. One of the problems for identifying the first stages of domestication is the difficulty to clearly differentiate guinea fowl bone remains from other species of wild Galliformes such as Francolins or of domesticated Galliformes such as chickens (MacDonald, 1992). In the same way, it is very difficult to distinguish whether the remains belong to wild or domestic individuals (MacDonald & MacDonald, 2000; Marshall, 2000), even if *N. m. galeata* has been considered to be the main parent subspecies of domesticated guinea fowl (Blench, 2000). From the 16th century, the Portuguese and Spanish reintroduced guinea fowl in Europe (already known during Greek and Roman antiquity, but disappeared with the collapse of the Roman Empire) from the west coast of Africa (Belshaw, 1985). Then, it has been spread in America and worldwide. Thus, the current domestic populations would come from *N. m. galeata*, which agrees with our results. In a previous mtDNA analysis on domestic populations from Nigeria, Kenya and China, Adeola et al. (2015) also showed a great proximity between African populations and individuals collected in China. This reinforces the idea of a common main origin for domestic guinea fowl, although without excluding the possibility of a secondary domestication, particularly from the capture of wild individuals and their integration into domestic livestock as is done in Kenya (Nyaga, 2007). Further sampling and subsequent analyses are needed to document better the origin and the process of domestication of guinea fowl, involving the different subspecies.

Because guinea fowl are roaming freely around farms, they are likely to meet and mate with wild relatives as has been demonstrated for chicken in Vietnam (Berthouly et al., 2009) and India (Kanginakudru, Metta, Jakati, & Nagaraju, 2008). Our sampling in Burkina Faso involved both wild and domestic populations (Figure 1), since samples of the two domestic populations (SDA‐t and SKO‐t) have been collected near the wild stock YAB‐w. Principal component analysis exhibits a clear differentiation between the wild samples and the domestic ones, thus suggesting a low level of admixture occurring that we can detect by our methods. A similar result was obtained by Weimann et al. (2016) in their study of the genetic diversity (microsatellites) of guinea fowl in Sudan, where the wild population appeared clearly differentiated from the domestic populations. By contrast, in a study of wild guinea fowl in South Africa, Walker et al. (2004) highlighted the presence of some domestic or hybrid individuals, within the wild populations in KwaZulu‐Natal. Since the 1980s, natural guinea fowl populations of this province have experienced a sharp decrease. To reinforce natural populations, restocking operations have been carried out with domestic animals (*N. m. galeata*), allowing contact and reproduction between wild and feral animals. Such a situation is neither reported for Burkina Faso nor Sudan.

The wild populations exhibit larger diversity and effective population size than the domestic ones. Same results were previously observed in other domestic species with living wild ancestors, such as chicken (Berthouly et al., 2009; Kanginakudru et al., 2008) or pig (Herrero‐Medrano et al., 2013; Rodrigáñez et al., 2008). Among the domesticated populations, genetic diversity is often higher in nonmanaged or preserved populations than in standard breeds or commercial lines (Berthouly et al., 2008; Granevitze et al., 2007; Leroy et al., 2012). Kayang et al. (2010), using microsatellite markers, observed a similar discrepancy when comparing traditional guinea fowl populations from Benin and Ghana with Japanese commercial stocks. We found the same tendency but at a lower extent. This must be related to the sampling procedure defined for the breeders’ lines. Samples from Beghin have been collected after the disappearance of the lines from this breeder's company. Thus, we have collected, both for DNA collection and semen cryopreservation, samples from the last living commercial flock, which was not a pure selected line but an intercross. Samples from the two other companies (Grimaud Frères Sélection and Galor, today merged under the Galor brand name within the Groupe Grimaud) represented pure lines that were pooled for the analysis. The pooled data represented ten lines (two to four individuals/lines) for Galor and four lines (five individuals/line), for Grimaud Frères Sélection. Thus, these results represent the genetic diversity available in these breeding companies.

Several domestication regions detected contained genes, among which some have interesting features. The small 21 kb domestication region at position 7.3 Mb on chromosome 7 (FiguresS2 and S6A, Tables S6 and S10) contains *PAPPA2* as the only gene. Moreover, 27 SNPs within a 20 kb portion of the gene have extreme allele frequency differences between the wild and the domesticated populations and one has a missense effect. Interestingly, this narrow peak can be attributed to the African traditional populations from Burkina Faso and Benin, whereas in the European populations, the selection signature encompasses a much wider region, of 219 kb (Figure S2 and Table S6). A narrow selection signature suggests an ancient event, whereas a larger region suggests recent selection. One hypothesis explaining this observation could be that a first round of selection might have happened when guinea fowl was domesticated in Africa and that a second round took place, either affecting another gene nearby or one of the remaining haplotypes in *PAPPA2*, after importation of the species in Europe. *PAPPA2*, known as Pregnancy‐Associated Plasma Preproprotein‐A2, has long been used as a marker of foetal genetic disorders (Wang et al., 2009). It is a protease which cleaves insulin‐like growth factor‐binding proteins *IGFBP‐3* and *IGFBP‐5* and is thus one of the modulators of IGF‐I bioavailability (reviewed in Fujimoto, Hwa, and Dauber (2017)). *PAPPA2* was reported as a strong candidate for a QTL affecting body size in mice (Christians, Hoeflich, & Keightley, 2006) and was also among the 180 loci detected in a GWAS on human adult height performed on close to 200,000 individuals (Lango Allen et al., 2010). Finally, effects of *PAPPA2* on female reproduction performances (Hawken et al., 2012) and also on adult height (Bouwman et al., 2018) were observed in cattle. Thus, *PAPPA2* is involved in genetic control of body size in three vertebrate species. The finding of *PAPPA2* domestication signature suggests that body size and meat production, rather than egg production, were motivation for guinea fowl domestication.

Interestingly, *SLC41A2*, a magnesium transporter (Sahni, Nelson, & Scharenberg, 2007) on chromosome 1 around 55.0 Mb, was also found in our data in a region selected after importation into Europe. Although it only contains eight SNPs with extreme frequency differences, it is worth noting that it was included in one of the eight regions showing a sweep signature in all four breeds investigated in a sequencing study in cattle (Boitard, Boussaha, Capitan, Rocha, & Servin, 2016) and might therefore be related to selection after domestication of these species.

Other regions show very strong evidences of selection but include a large number of potential candidate genes. For instance, the largest of the two domestication regions on chromosome 8 is 175 kb long at 11.1–11.3 Mb and is detected by both methods. It is flanked by two annotated genes (*TMLHE* and *VAMP7*) and contains 12 genes in total, three of which (*TMLHE*,* SPRY3* and *VAMP7*) have gene names in the annotation and a fourth, *LOC110403465*, has a missense polymorphism and the description ‘*EDNRB*‐like’ (Figure S6B). These 12 genes contain a total of 148 SNPs with extreme frequency differences between the wild and the domesticated populations and three more unknown genes very close at 11.5 Mb on chromosome 8 contain 40 such SNPs. Evidence from the literature suggests that *TMLHE* and *VAMP7* could play a role in domestication through modulation of behaviour. *TMLHE* is the first enzyme in the carnitine biosynthesis pathway, and *TMLHE* deficiency causes regressive autism symptoms that can be improved via carnitine supplementation (Ziats et al., 2015). It is worth noting also that a region containing a gene involved in autism in human was detected when comparing two lines of quail divergently selected on social behaviour (Fariello et al., 2017). Severe mutations in *TMLHE* cause extreme phenotypes, such as seen in human and model organisms, and it would not be surprising that a polymorphism in such a gene causing milder phenotypes could reside in regulatory region situated for instance in the promoter or in introns. At the 3′ end of this selected region, *VAMP7* codes for a protein which localizes to late endosomes and lysosomes and is involved in the fusion of transport vesicles to their target membranes; a knock‐out of *VAMP7* exon 3 caused reduced brain weight and anxiety in mice (Danglot et al., 2012).

Close to *VAMP7*,* EDNRB* is a gene expressed in melanocytes which derive from the neural crest, and for this reason, *EDNRB* is particularly mentioned by Wilkins, Wrangham, and Fitch (2014), who propose that an alteration of the development of neural crest cells would explain various phenotypes found in domesticated animals and not in their wild ancestors. Some neural crest‐related genes were also found to be associated with selection signatures in the domestic cat (Montague et al., 2014), whereas the universality of this theory was critically examined by Sánchez‐Villagra, Geiger, and Schneider (2016) who pointed out the lack of data in many species. Additional measures are thus needed to verify whether other domestication traits, such as tameness, are also modified in domestic guinea fowls, which could then validate the neural crest domestication theory in a bird. Another possible explanation could just involve selection on pigmentation. Considering that several coding mutations of *EDNRB2* have been associated with extended white spots or extremely diluted plumage colour in some breeds of domestic chickens (Kinoshita et al., 2014), we performed a careful analysis of the pictures available for the guinea fowls sampled in West Africa. Whereas all wild guinea fowls exhibited a dark skin on the whole body and a regularly spotted plumage with small white spots (Figure S7), all domestic guinea fowls exhibited large patches of white skin on the head, and white or yellow areas on the shanks. In addition, 16 out of 31 birds exhibited large white spots on the belly feathers and several wing feathers were fully white (Figure S8), mimicking the mottled phenotype encountered in chickens carrying an *EDNRB2* mutation (Kinoshita et al., 2014). Interestingly, five of 31 birds exhibited an extremely diluted phenotype from pale grey to full white, where a ghost spotting pattern could be distinguished (Figure S9), mimicking the mo*w mutation of *EDNRB2* described in a full‐white Japanese breed of chickens by Kinoshita et al. (2014). The proportion of domestic guinea fowls exhibiting extended white patches, or extreme dilution, was higher in Burkina Faso (12/16) than in Benin (4/15). Since the white‐spotting mutations associated with mutations in *EDNRB2* are recessive in chickens, it is likely that some domestic guinea fowls do not exhibit an extended white phenotype while being heterozygous carriers of a recessive mutation in *EDNRB*. Considering the association of *EDNRB* with extension of white in the plumage, we also searched for a possible advantage of such plumage pattern. In Benin, a cultural value has been proposed to be associated with white‐spotting in chickens, including association to luck, wealth or peace (Chrysostome, Houndonougbo, Houndonougbo, Dossou, & Zohoun, 2013; Faustin et al., 2010). By analogy, the domestication signature found on *EDNRB* in domestic guinea fowls could just indicate a preference of farmers for an extended white plumage colour, without any proven relationship with another biological function. In conclusion, the domestication signature on chromosome 8 involves three genes (*TMLHE*,* VAMP7* and *EDNRB*), the three of them possibly important drivers of the domestication signature through different biological mechanisms, without excluding, at this stage, the neural crest theory.

This first study of guinea fowl genetic diversity was based on Pool‐seq data, which is a very cost effective approach for estimating population allele frequencies genome‐wide (Schlötterer, Tobler, Kofler, & Nolte, 2014). We used specific statistical methods developed for this kind of data (pool‐hmm or popoolation). These methods account for the effects of sample size, coverage or sequencing errors and have been extensively validated using simulations. For the detection of positive selection, we looked for signatures at the level of genomic regions (with pool‐hmm and the local score), which both limits the influence of allele frequency estimation accuracy at each single SNP and exploits linkage disequilibrium information. However, we cannot exclude that our Pool‐seq approach, with limited sample sizes and coverage in most pools, had an impact on the results. For instance, focusing on a small part of the genome (Chromosome 8), we compared the estimations of Watterson *θ* obtained with raw data with those obtained after subsampling all populations and positions at a uniform coverage of 10× (Figure S10). Although these two approaches lead to the same general conclusion, we observed that two populations (GAL‐s and Gri‐s), characterized by a combination of higher coverage and sample size compared to all others, had a significantly higher diversity based on subsampled data. This suggests that the formula used in popoolation, which includes a correction term accounting for sample size and coverage, is slightly biased. This is not expected to occur, but we note that the simulations provided by the authors of this software focused on much higher values of these parameters than the ones considered here (Kofler, Orozco‐terWengel et al., 2011). Another potential drawback of Pool‐seq data may be to limit the detection power of selective sweeps with the population differentiation approach. Indeed, while the estimation of allele frequencies by pool‐hmm is expected to be unbiased, the variance of this estimation is increased by the stochastic contribution of one given individual to the pool at each position, compared to individual sequencing. This leads FLK to overestimate the amount of drift, thus reducing its capacity to detect selection events with increased allele frequency variance between populations. The pool‐hmm approach, on the other hand, has been shown by the authors to be as powerful as the equivalent HMM approach based on ideal true allele frequency data, for sample sizes as low as 25 alleles. Overall, we anticipate that further studies based on individual sequencing would refine our conclusions concerning guinea fowl domestication history, but not change them fundamentally.

In conclusion, we present here the first genome assembly of the guinea fowl and its utility in a selection signature study of domestication. Using the Galgal5 chicken genome as a reference and published comparative cytogenetic data has proven an efficient method for working at the chromosome level. The pooled whole‐genome sequencing approach has revealed the main features of domestication and selection in guinea fowl, for which the domestication scenario could be refined. We propose that the ancestors of the guinea fowls bred in Europe come from the traditional populations of guinea fowls of Western Africa, and furthermore, we show that body size, behaviour and plumage colour are likely to be the main motivations for domestication of this species. We also confirm that the gene pool of European guinea fowls is a limited subset of the genetic variation present in domestic guinea fowls of Africa. While some genes detected here were also found in similar studies performed in other species, a convergent role in their suspected involvement in the domestication process will require more comparative studies. We plan to sample more wild and traditional domestic populations from Africa, as well as domestic guinea fowls from other continents, to strengthen and refine the selection signatures discovered thus far.

## AUTHOR CONTRIBUTION

X.R., A.V. and M.T.B. conceived the project. A.V., M.T.B., G.‐K.D., V.Y.‐G., I.Y., J.B., D.G., C.B.‐S. and X.R. planned the selection of samples and data collection. A.V. coordinated the sequencing of the reference genome. W.W. supervised the sequencing and scaffold assembly and F.T.‐N. the annotation. A.V. and N.T. assembled the scaffolds into chromosomes. SB performed the genetic diversity and selection signatures analyses. S.B., A.V., M.T.B. and X.R. wrote the manuscript, and all the authors participated in the discussion. All authors read and approved the final manuscript.

## Data Availability

The genome assembly and its annotation are publicly available at the NCBI genome database https://www.ncbi.nlm.nih.gov/genome/14094 under the name of NumMel1.0 (accession GCA_002078875.2). Raw read sequences (fastq files) generated in the pooled population sequencing have been deposited in the Sequence Read Archive (SRA) at www.ncbi.nlm.nih.gov/sra under the project accession PRJNA496587. The scripts used for the diversity analysis and intermediate files with the estimation of allele frequencies in the populations have been deposited in the zenodo archive: https://doi.org/10.5281/zenodo.2553884.
