# Supplementary material for: A guinea fowl genome assembly provides new evidence on evolution following domestication and selection in galliformes
Source: Mol Ecol Resour. 2019 May 5;19(4):997–1014. doi: 10.1111/1755-0998.13017 (PMC6579635; doi:10.1111/1755-0998.13017)
Supplement: Supplementary file 1 [file MEN-19-997-s001.zip › men13017-sup-0001-FigS1-S10.pdf]

A guinea fowl genome assembly provides new evidence on evolution following domestication and selection in galliformes

Alain Vignal, Simon Boitard, Noémie Thebault, Guiguigbaza-Kossigan Dayo, Valentine Yapi-Gnaore, Issaka Youssao, Cécile Berthouly-Salazar, Nóra Pálinkás-Bodzsár, Daniel Guémené, Francoise Thibaud-Nissen, Wesley C. Warren, Michèle Tixier-Boichard and Xavier Rognon

Supplementary figures

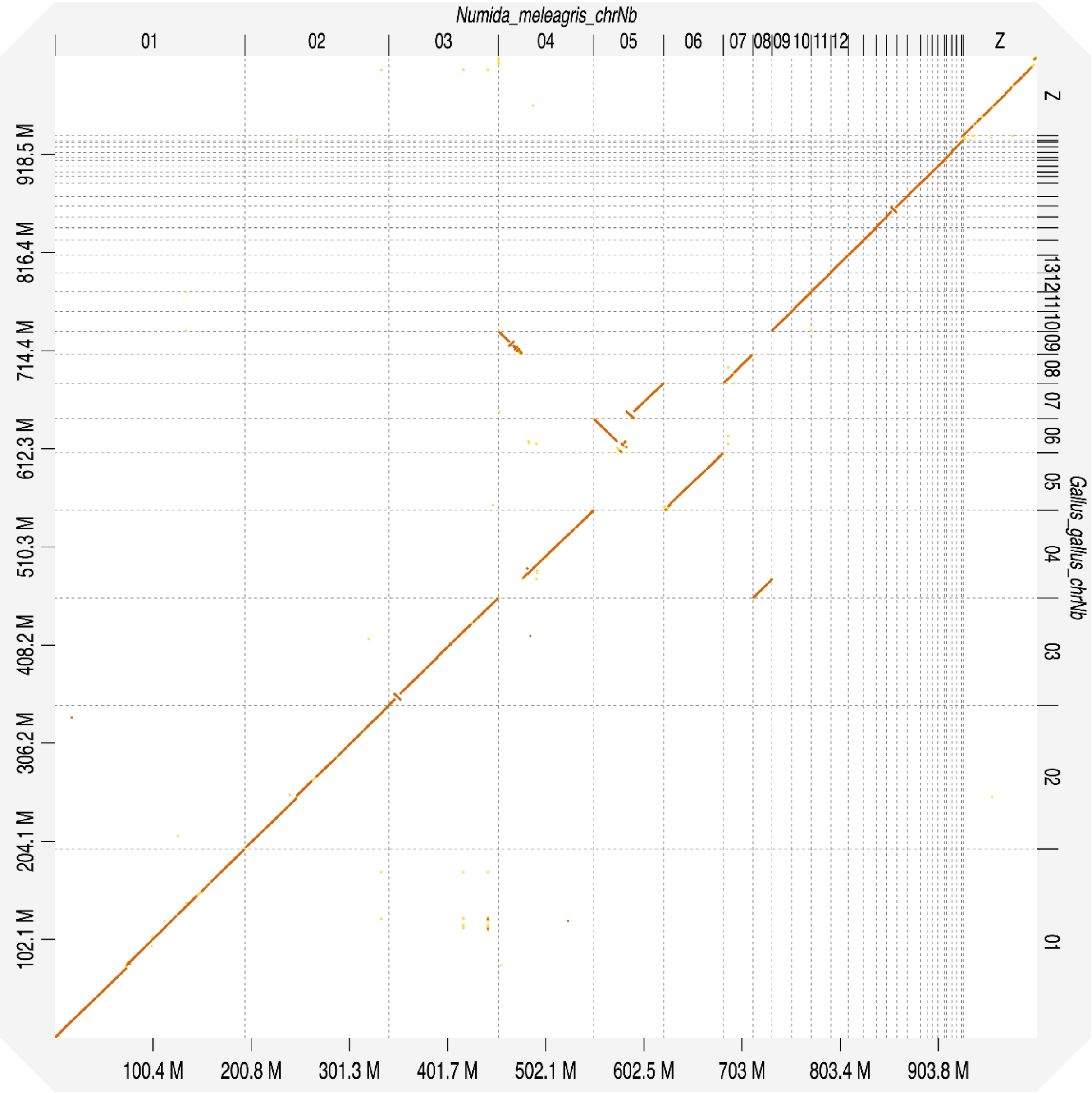

Figure S1. Alignment of the guinea fowl and chicken genomes. The genomes of the chicken and guinea fowl are aligned using D-Genies available at http://dgenies.toulouse.inra.fr/ (Cabanettes and Klopp 2018).

Reference:
Cabanettes F, Klopp C (2018) D-GENIES: dot plot large genomes in an interactive, efficient and simple way. PeerJ 6:e4958

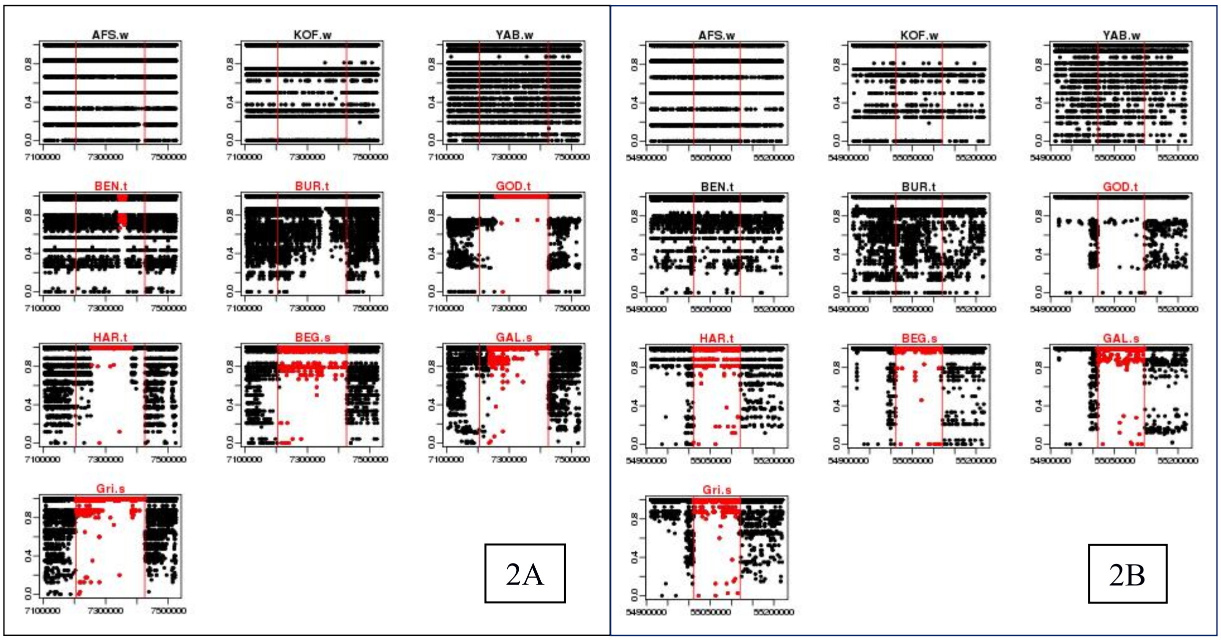

Figure S2. Genetic diversity in two candidate regions under selection. The first region (2A) is located on chromosome 7 and is related to domestication, the second one (2B) is located on chromosome 1 and is related to importation into Europe. Ten panels, corresponding to the 10 analysed populations, are shown for each region. These panels represent the frequency of the major allele (y axis) for all bi-allelic variants within the region, as a function of their genomic position (x axis). Red points indicate the region that is detected by Pool-HMM in each population, and vertical red lines indicate the union of detected regions when combining all populations under selection. In the two domestic African populations (BEN-t and BUR-t), a selection signature is detected in the region related to domestication (on the left), but not in the region related to the importation to Europe.

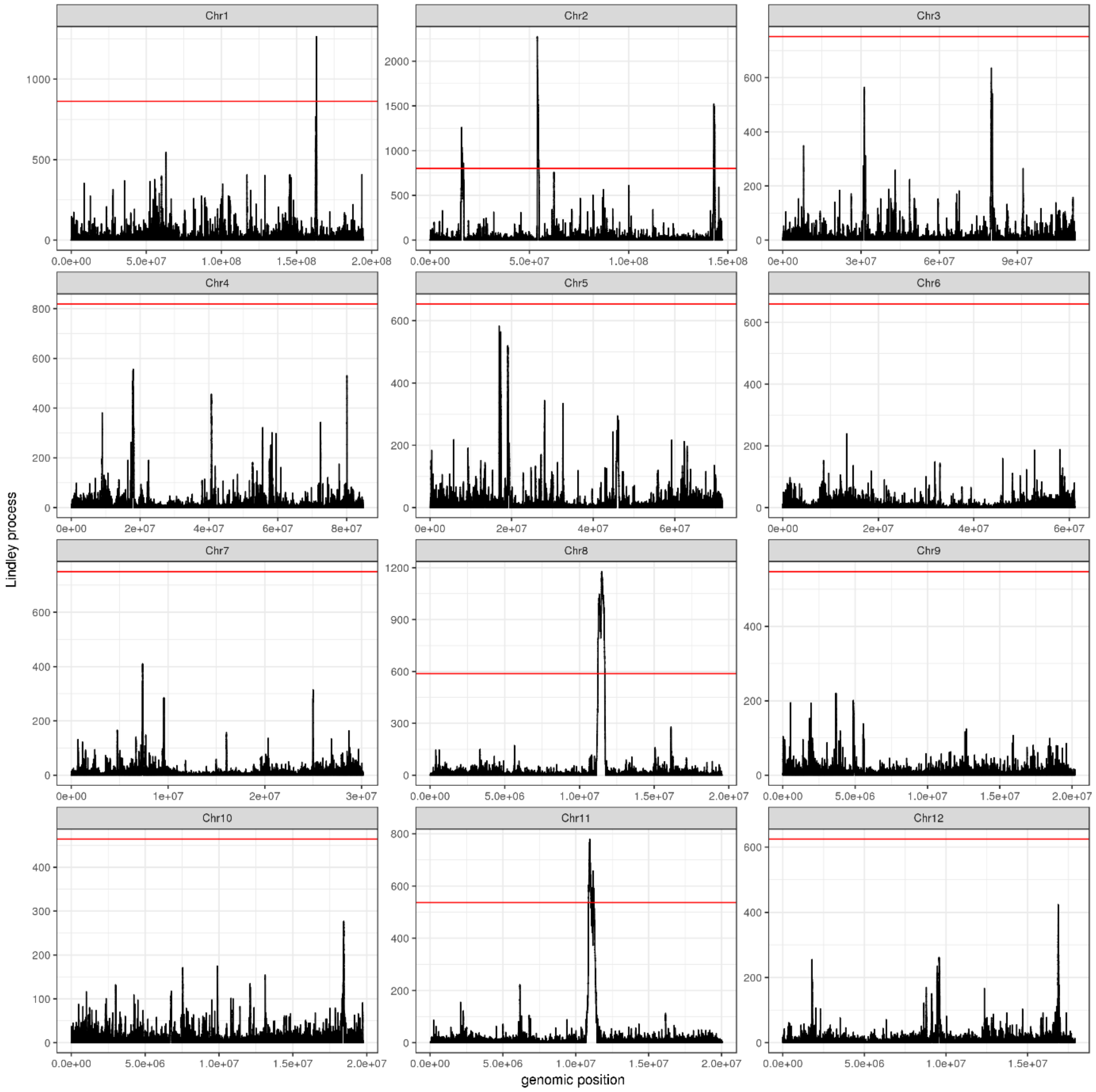

Figure S3. Chromosomes 1-12. Lindley process, which cumulates scores at consecutive variants (Fariello et al 2013). The horizontal red line indicates a chromosome-wide type I error rate of 5%.

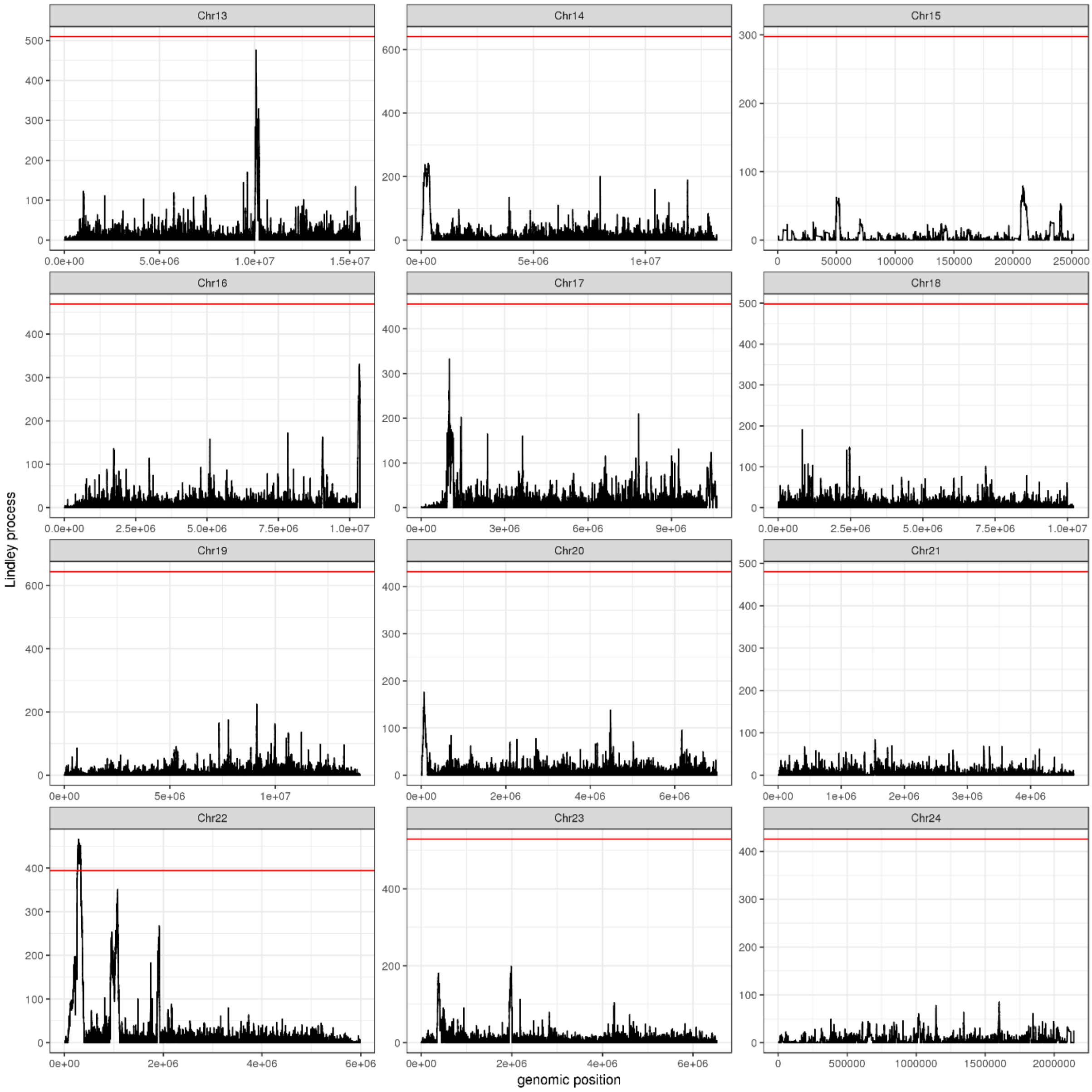

Figure S4. Chromosomes 13-24. Lindley process, which cumulates scores at consecutive variants (Fariello et al 2013). The horizontal red line indicates a chromosome-wide type I error rate of 5%.

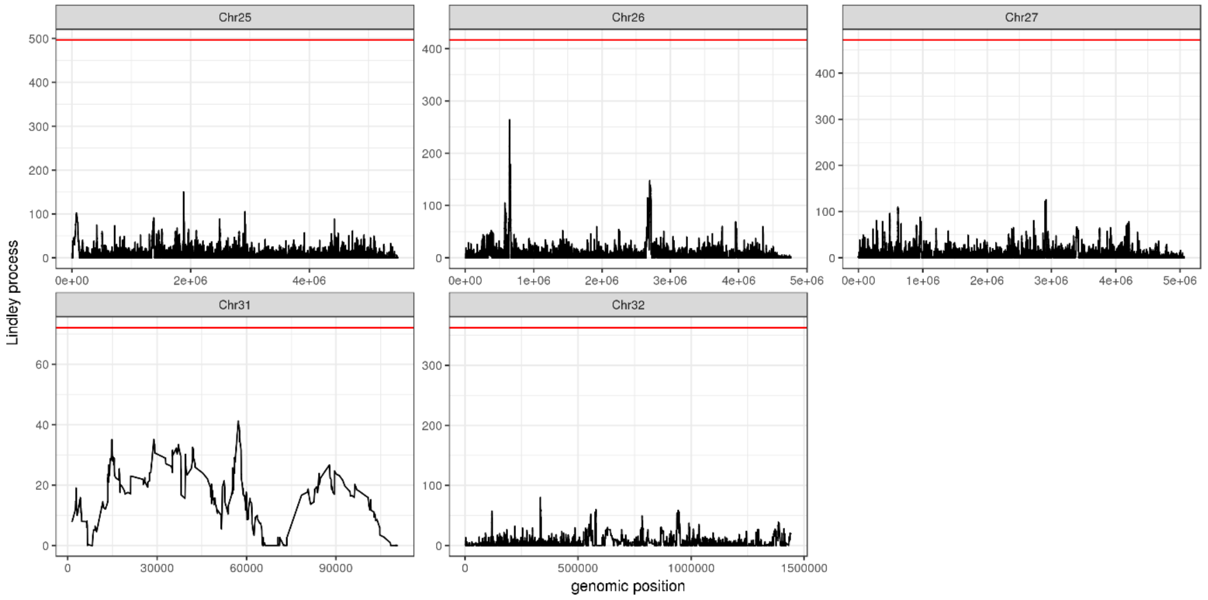

Figure S5. Chromosomes 25-27; 31-32. Lindley process, which cumulates scores at consecutive variants (Fariello et al 2013). The horizontal red line indicates a chromosome-wide type I error rate of 5%.

A

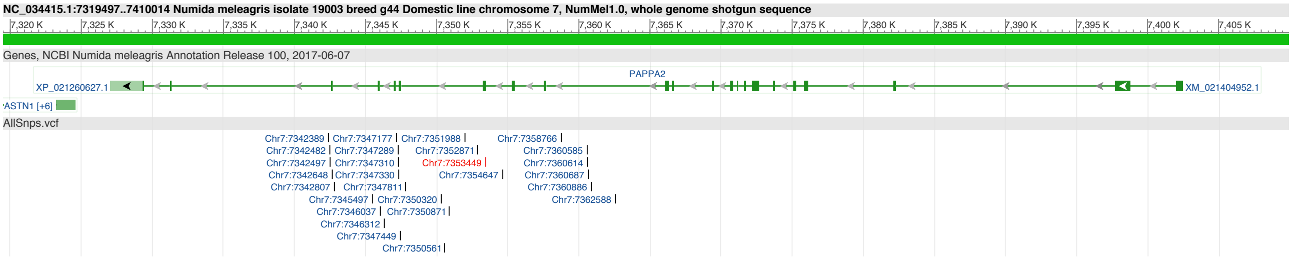

B

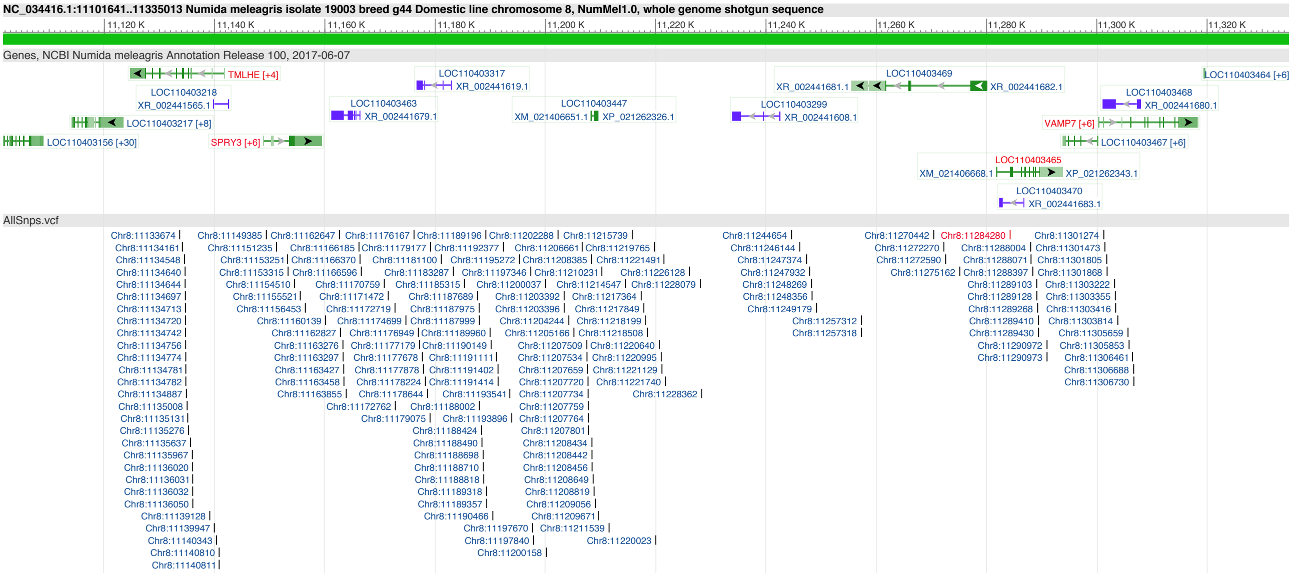

Figure S6: Examples of two regions with highly differentiated SNPs. Screenshots generated in the NCBI Genome Data Viewer, with custom tracks showing the SNPs with high differentiation between population (https://www.ncbi.nlm.nih.gov/genome/gdv/). A: SNP having extreme allele frequencies differences between the wild and domestic populations in the *PAPPA2* gene. The SNP responsible for a missense polymorphism is indicated in red. B: SNPs having extreme allele frequencies differences between the wild and domestic populations in the selected region on chromosome 8. The four genes discussed in the main text, including *LOC110403465* the *EDNRB-like* gene and the SNP responsible for a missense polymorphism, are indicated in red.

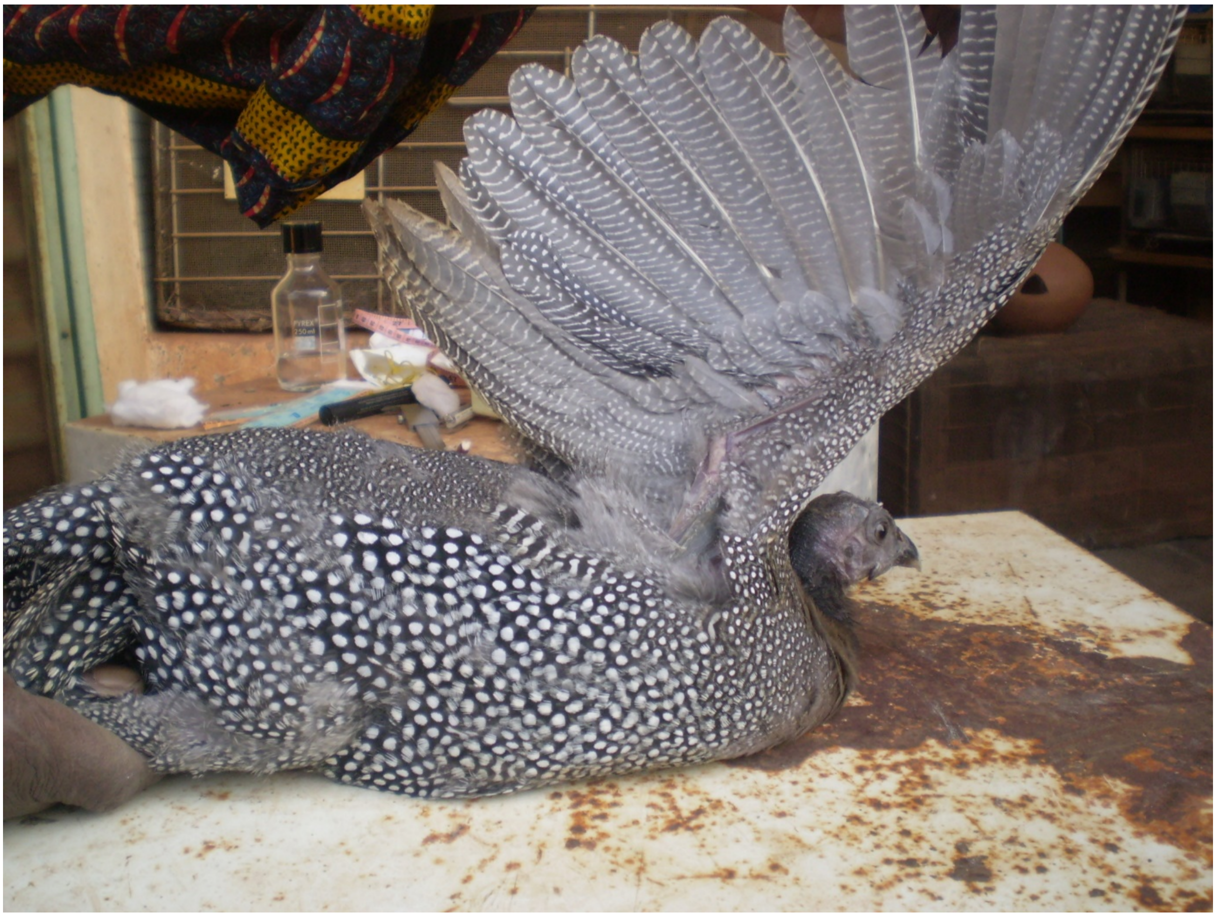

Figure S7. Wild Guinea fowl (collected at Yabé, Burkina Faso)

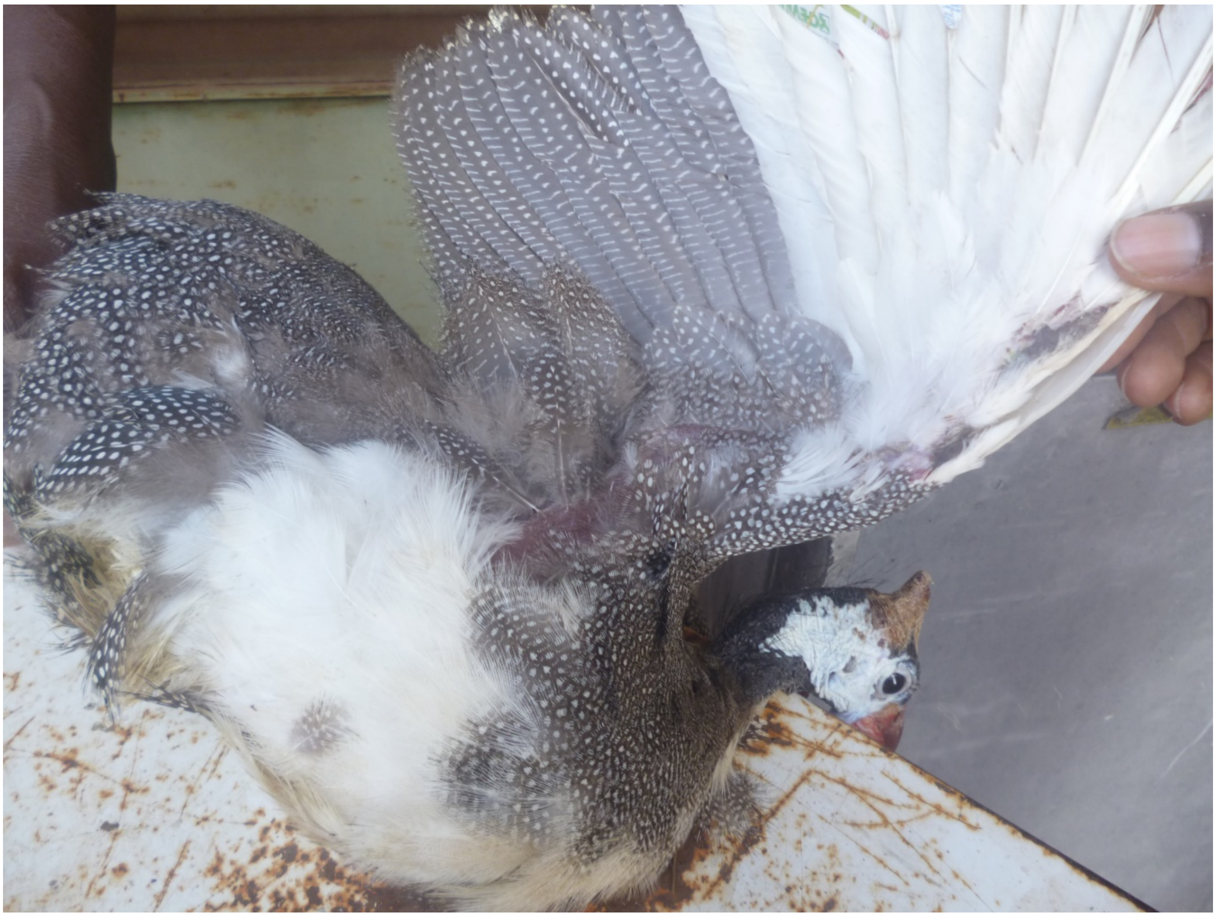

Figure S8. Domestic guinea fowl (collected at Dori, Burkina Faso) with a white-spotted plumage showing extended white patches and white-sided head.

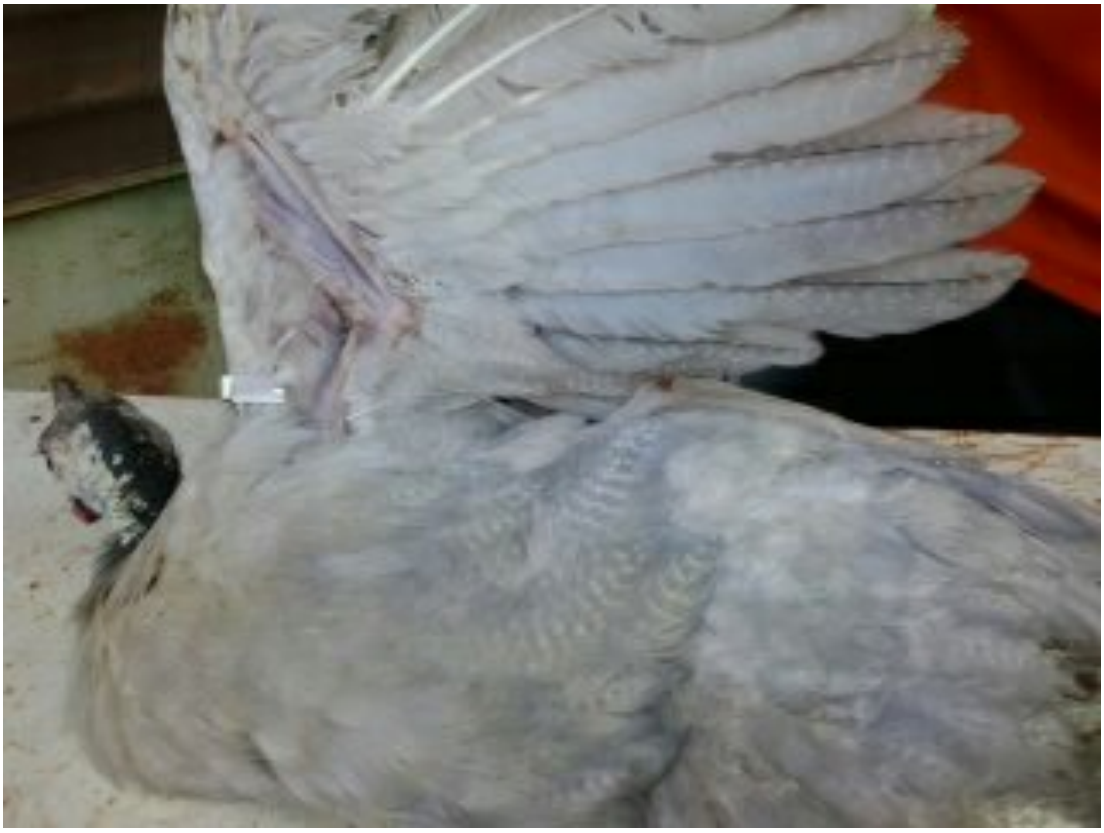

Figure S9. Domestic guinea fowl (collected at Parakou, Benin) with a pearl-grey plumage color and a visible diluted pattern, the head is also white-sided.

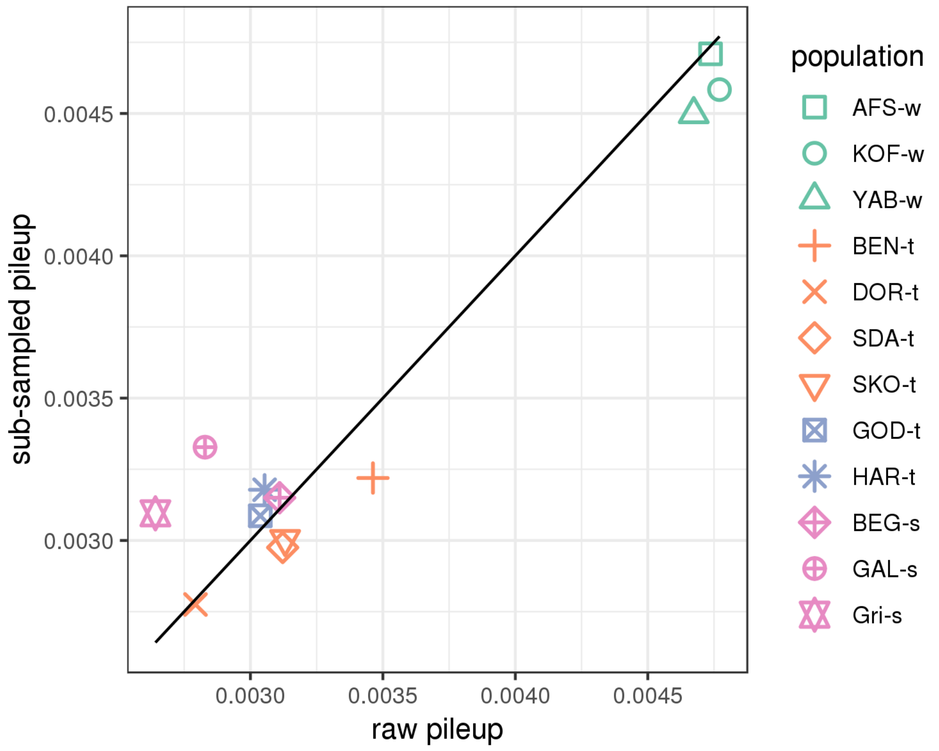

Figure S10. Influence of sub-sampling reads on the estimation of Watterson θ. Watterson θ values were calculated by using all reads (x-axis) or by sub-sampling all populations at uniform 10 X coverage, for chromosome 8 (y-axis).
