# Supplementary material for: A guinea fowl genome assembly provides new evidence on evolution following domestication and selection in galliformes
Source: Mol Ecol Resour. 2019 May 5;19(4):997–1014. doi: 10.1111/1755-0998.13017 (PMC6579635; doi:10.1111/1755-0998.13017)
Supplement: Supplementary file 1 [file MEN-19-997-s001.zip › men13017-sup-0004-Supinfo2.zip!/ScriptsGenoAssembly/README.pdf]

Supplementary data to the guinea fowl assembly and diversity analysis: scripts for guided chromosome assembly using the chicken reference

29 january 2019

Assembling guinea fowl scaffolds by alignment to the chicken assembly

Introduction

This section describes the scripts used for the guided assembly of the guinea fowl scaffolds into chromosomes, by using the Galgal5 chicken genome as a reference. The scaffolds were obtained by assembling the Illumina reads with the ALLPATHS2 assembler (see main text). Each scaffold in the fasta format is aligned using the Lastz software to Galgal5. The output .maf files are used for determining the orientation, start and stop and positions of the aligned scaffolds on the chicken chromosomes. This information is used to concatenate the scaffolds into a chromosome sequence using biopython. These scripts were used to automate the process as much as possible and to produce intermediate graphs to help taking decisions on thresholds to use for the selection of scaffolds or for deciding whether some scaffolds had to be splitted, when suspected of being chimeric.

Aligning guinea fowl scaffolds on Galgal5 with LastZ

Separating guinea fowl scaffolds into fasta files: one per scaffold

Guinea fowl scaffolds fasta file format:

```
>Scaffold0
TATTTAGTAAATTAGTTTGTTTCTCCTCAGATTGTTGCCGCTGTTCTTTGCTCTCAGGGC
CATCTCCCT...
>Scaffold0
ATGATTTAGGTGAAATTGATGTCTGCAAGTACACGGACTCTAATGGGATGCAACAATAAG
TGCTGATGTTATTGCTAAGCCACTCTCCATCATCTTTGAAAGGCTGTAGAGAATAAGAGA
AGTGTGACT...
```

sepScaffNum.py

```
* input: multifasta file
* output: one sequence per file
```

Calling LastZ: aligning guinea fowl scaffolds on Galgal5 and producing output for plotting

- The script call_alignscaffolds.bash will loop over all the scaffoldXX.fa files, to call the align_scaffolds.bash script and send the jobs on a sge cluster.
- align_scaffolds.bash will call the three scripts:
    - scriptLastzV2.sh for performing the alignments with Lastz
    - modifyDatFileScaffold.py for converting the output into csv format
    - PlotScaffold.R for plotting the alignments in R
- The plots will then be examined to help setup parameters for performing the assembly into chromosomes

call_alignscaffolds.bash

```
* Script for looping over all scaffolds
* Calls align_scaffolds.bash
```

align_scaffolds.bash

```
* Called by call_alignscaffolds.bash
* Calls scriptLastzV2.sh
* Calls modifyDatFileScaffold.py
* Calls PlotScaffold.R
```

scriptLastzV2.sh

```
* Called by align_scaffolds.bash
* Does the Lastz alignments
```

modifyDatFileScaffold.py

```
* Called by align_scaffolds.bash
* Generates a csv file for plotting scaffold alignments to chicken chromosomes
in R
```

PlotScaffold.R

```
* Called by align_scaffolds.bash
* Plots the alignments of the scaffolds onto the chicken chromosomes
```

Visual inspection of alignments to choose filtering parameters

- Most scaffolds have a main alignment to a chromosome, plus some less significant alignments.

- Filtering on alignment scores eliminates most non-significant alignments
- Some scaffolds may retain significant alignments to more than one chromosome. There are two ain causes for this:
  - Chimeric scaffolds: these will be separated into two (or more) scaffolds: scaffoldXXa and scaffoldXXb.
  - Small scaffolds for which alignments will be too short for unambigous chromosome assignment.

Figure 1: Length distribution of Lastz alignments of scaffold0 on Galgal5 chromosome 2.

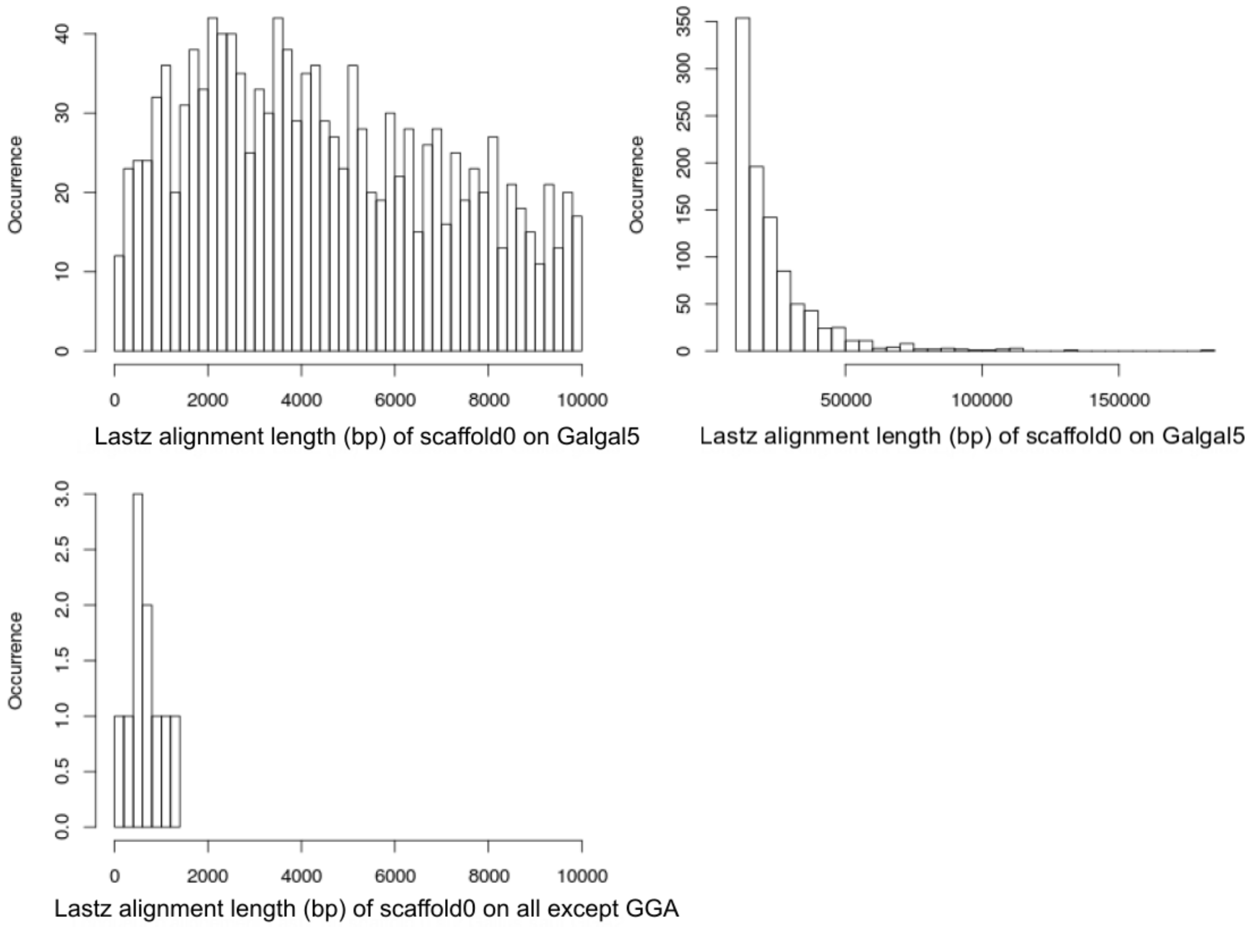

Length distribution of Lastz alignments of scaffold0 on Galgal5. Top left: alignments smaller than 10 kb; top right: alignments longer than 10 kb; bottom: alignments on all the genome, except chicken chromosome 2 (GGA2). Alignments on chromosomes other than GGA2 are very short and can therefore be considered as non-specific.

Figure 2: Alignment of Numida scaffold0 on Gallus chromosome 2.

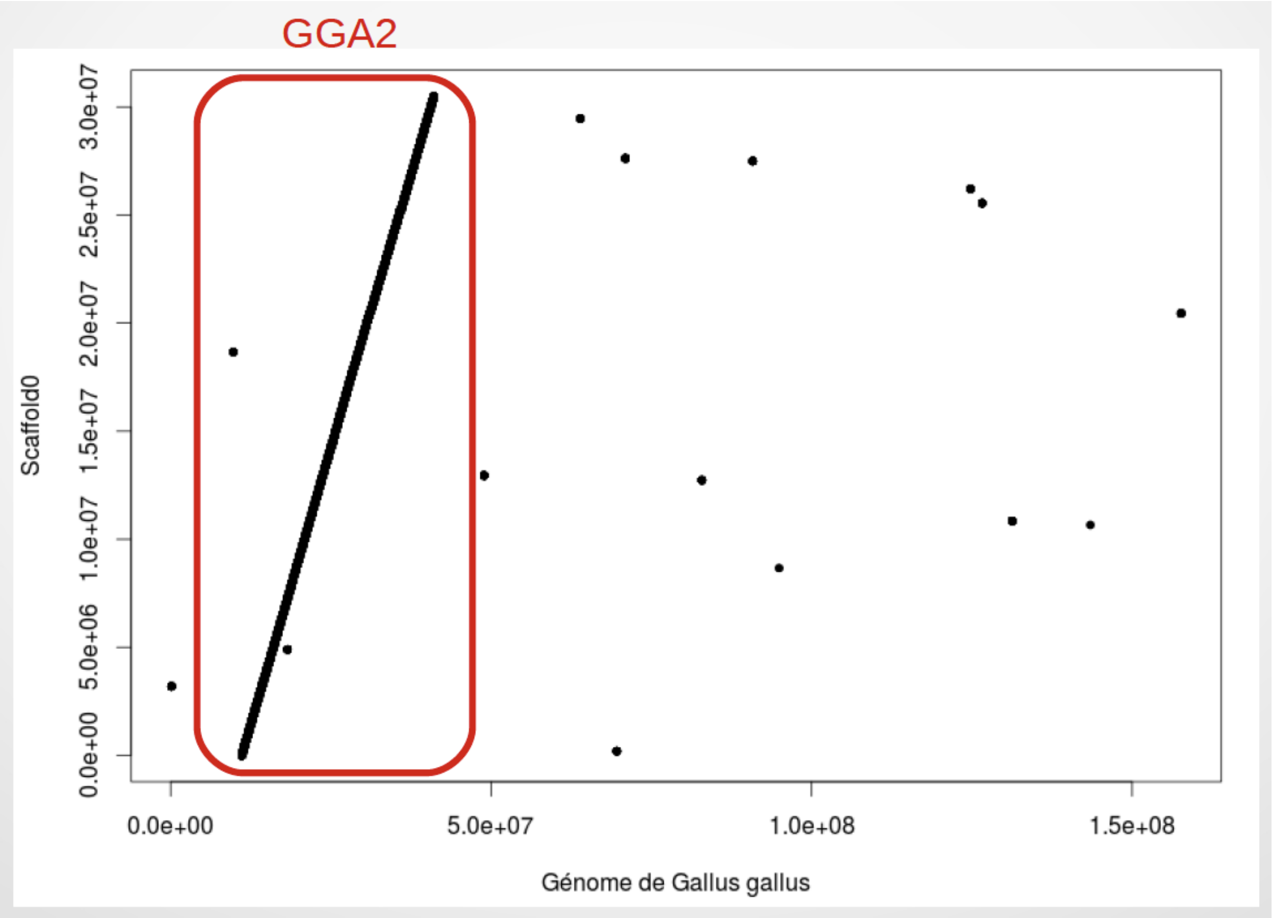

Alignment of Numida scaffold0 on Gallus chromosome 2. The diagonal represents the main alignment and the dots represent small non-specific alignments. The presence of these small non-specific alignments complicates the determination of the real start and end positions of the guinea fowl scaffold alignments on the chicken chromosomes by a direct analysis of the Lastz .maf output file.

Figure 3: Filtering for guinea fowl scaffolds with unambigous assignation to chicken chromosomes.

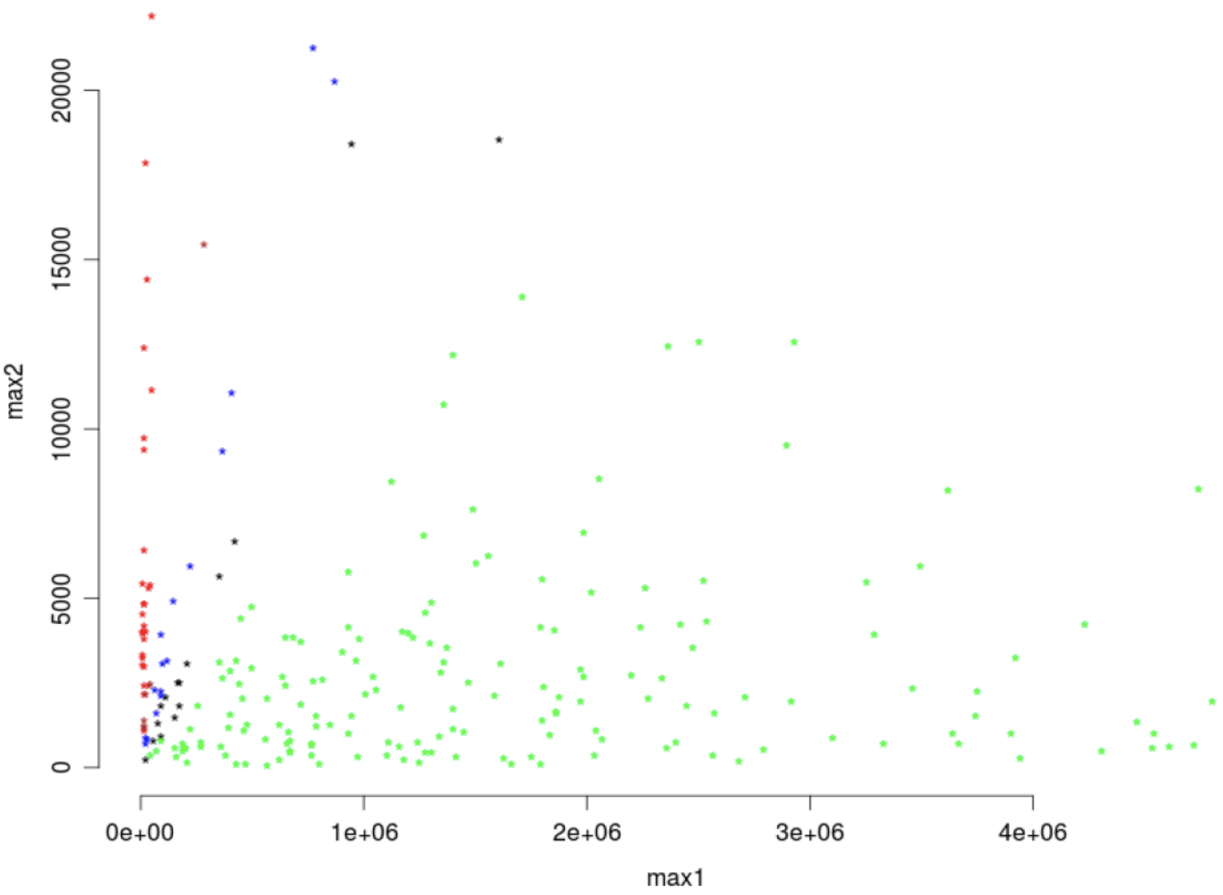

Filtering for guinea fowl scaffolds with unambigous assignation to chicken chromosomes, based on alignment metrics in from .maf Lastz alignment files. Max1: sum of alignment lengths to most significant chicken chromosome. Max2: sum of alignment lengths to second most significant chicken chromosome. Colors correspond to different values of the max2/max1 ratio: green = 0 - 0.01; black = 0.01 - 0.02; blue = 0.02 - 0.05; red = 0.05 - 1.00. Scaffolds having an alignment longer than 5 Mb to a single chromosome are not shown. Based on this graph, the threshold for selecting scaffolds for the assembly was set to 0.01. All scaffolds under this threshold were assigned to the “chrUnplaced.fa” file. unknown“.

Figure 4: Scaffolds aligned to GGA1 (left: A and C) and GGA3 (right: B and D).

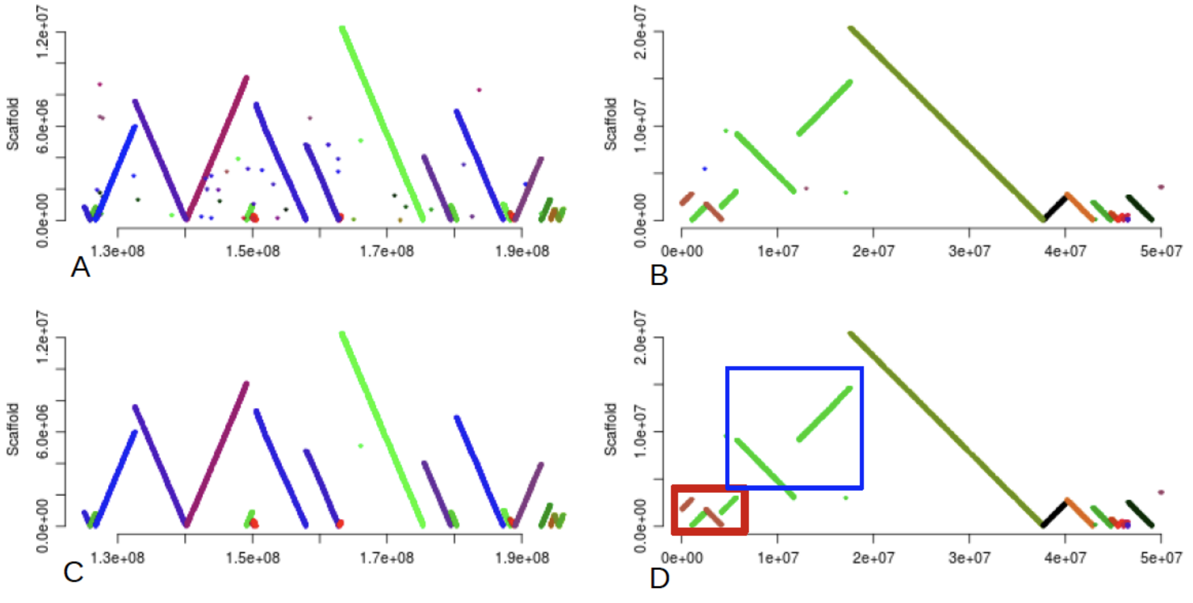

Scaffolds aligned to GGA1 (left: A and C) and GGA3 (right: B and D). Top (A and B): for most scaffolds, there are main alignments (diagonals) and non-specific alignments (dots). Bottom (C and D): filters on the alignment scores allow to remove the non-specific alignments. This step is essential for determining the real start and stop positions on guinea fowl scaffolds on chicken chromosomes. Red and blue boxes represent chicken-guinea fowl rearrangements detected by the non-linear alignment of the scaffolds.

Assembling scaffolds into chromosomes

- scaffoldXX.dat files produced by the scriptLastzV2.sh, once filtered (see above) are concatenated by chromosome into GGAX.dat files
- These GGAX.dat are input files for the script PipelineAssemblyScaffolds.bash
- Script PipelineAssemblyScaffolds.bash
  - Calls different scripts according to specific cases: AssemblyOneScaffold.sh ; AssemblyManyScaffolds.sh … are called according to each specific case.
  - Input: ChrParticularity.txt file
    - A list in the form: ChickenChromosome Particularity
      - Where particularity is:
        - ‘OneScaffold’: only one guinea foiwl scaffold assigned to the chicken chromosome
        - ‘ManyScaffolds’, several scaffolds assigned to the chromosome
        - ‘Crossing’: one or both coordinates of a scaffold are within the boundaries of another scaffold.
        - ‘BackgroundNoise’: filtering of .maf file needs to be improved to determine unambigously the scaffold boundaries
        - ‘Remove’: removes part of the alignment, which will be put in a “chrXX.unlocalised.fa” file.
        - etc. (see categories in the case statement in the script PipelineAssemblyScaffolds.bash)
  - Writes fasta files of assembled guinea fowl chromosomes and csv files for plotting alignment in R, for monitoring progress.

Main script: PipelineAssemblyScaffolds.bash

```
* Calls the various assembly scripts according to cases
```

Assembly scripts called by PipelineAssemblyScaffolds.bash

AssemblyOneScaffold.sh

```
* When only one scaffold corresponds to a chicken chromosome
```

AssemblyManyScaffolds.sh

```
* When several scaffolds are assigned to a chromosome
* Calls AssemblyManyScaffolds.py
```

AssemblyJustCrossing.sh

```
* When several scaffolds are assigned to a chromosome and a scaffold is insert
ed into another
* Calls AssemblyCrossingManyScaffolds.py
```

AssemblyBackgroundNoiseManyScaffolds.sh

```
* Calls AssemblyManyScaffolds.py
    * Input is a dat file modified by AssemblyBackgroundNoise.sh
```

AssemblyBackgroundNoiseCrossing.sh

```
* Calls AssemblyCrossingManyScaffolds.py
    * Input is a dat file modified by AssemblyBackgroundNoise.sh
```

AssemblyBackgroundNoise.sh

```
* For removing small non-specific alignments from Lastz output dat files
* Calls
    * AssemblyBackgroundNoise.py
    * modifyDatFileV7.py
```

AssemblyRemoveBases.sh

```
* Calls
    * AssemblyRemoveBases.py
    * AssemblyManyScaffolds.py
```

Diversity analysis

Mapping fastq files and generation of pileup files

Data

- DNA for each population is pooled, then one library is sequenced in several Illumina lanes.
- Fastq files are then in the form: population_library_lane_R1.fastq.gz and population_library_lane_R2.fastq.gz
- For instance: Gri-s_GTGAAA_L002_R1.fastq.gz and Gri-s_GTGAAA_L002_R2.fastq.gz
- With R1 and R2 containing the set of read pairs

Generic scripts for mapping all fastq files present in a directory

Script Maptest.bash for looping

```
* Maptest.bash will call the mapping.sh script for all samples in the director
y
```

mapping.sh

```
* Script called by Maptest.bash
```

Merging data

Script Mergetest.bash for looping

```
* Mergetest.bash will call merging_realigning.sh
* merging_realigning.sh will merge bam files from a same population, that were
sequenced in different lanes and perform the realignment around indels
```

merging_realigning.sh

```
* merging_realigning.sh called by Mergetest.bash
* Will produce the final pileup file for diversity analysis by Pool-HMM
```

The diversity analysis is then done on the pileup files with the scripts found in the supplementary file GuineaFowlDivScripts.tar.gz
